# Cellular and Molecular Effects of Targeting the CBP/β-Catenin Interaction with PRI-724 in Melanoma Cells, Drug-Naïve and Resistant to Inhibitors of BRAF^V600^ and MEK1/2

**DOI:** 10.3390/cells14211710

**Published:** 2025-10-31

**Authors:** Anna Gajos-Michniewicz, Michal Wozniak, Katarzyna Anna Kluszczynska, Malgorzata Czyz

**Affiliations:** Department of Molecular Biology of Cancer, Medical University of Lodz, 6/8 Mazowiecka Street, 92-215 Lodz, Poland; michal.wozniak@umed.lodz.pl (M.W.); kaska.kluszczynska@gmail.com (K.A.K.); malgorzata.czyz@umed.lodz.pl (M.C.)

**Keywords:** survivin, melanoma, CBP/β-catenin signaling, PRI-724, drug resistance

## Abstract

Targeted therapies, including treatment with inhibitors of BRAF^V600^ and MEK kinases, have improved outcomes in advanced melanoma. However, most patients relapse due to acquired resistance, underscoring the need for new drug targets. This study evaluated PRI-724, a CBP/β-catenin inhibitor, in patient-derived drug-naïve melanoma cells and their trametinib- or vemurafenib-resistant counterparts. While PRI-724 has demonstrated efficacy in preclinical models and clinical trials in different cancer types, its potential in melanoma has not been previously assessed. We found that treatment with PRI-724 downregulated survivin and other CBP/β-catenin target proteins, reduced invasiveness, and induced apoptosis in drug-naïve and trametinib- and vemurafenib-resistant cells. Trametinib-resistant melanoma cells showed the greatest sensitivity to PRI-724, indicating that CBP/β-catenin transcriptional activity may represent a new therapeutic vulnerability. Transcriptomic and immunoblotting analyses revealed the highest survivin levels in vemurafenib-resistant cells, which may underlie their reduced responsiveness to PRI-724. Bioinformatic analyses (TCGA and GSE50509) confirmed that a high survivin level predicts poor prognosis and reduced response to treatment. The results of the study point to the potential of PRI-724 as a chemotherapeutic agent for the treatment of melanoma. Its efficacy might depend on CBP/β-catenin transcriptional activity in melanoma cells, and further evaluation of this signaling with survivin as a biomarker is therefore warranted.

## 1. Introduction

Resistance to conventional chemotherapy and newly developed targeted therapies is characteristic of numerous cancer types, including melanoma. Melanoma, recognized as the most lethal skin cancer [1], is highly heterogeneous and demonstrates considerable interpatient variability in its genotypic and phenotypic features [2]. One of the most prevalent genetic alterations observed in melanoma is V600E in B-Raf murine sarcoma viral oncogene homolog B (BRAF), which largely contributes to constitutive activation of the RAS/RAF/MEK/ERK signaling pathway, known as the MAPK pathway. The advent of targeted therapies, including BRAF^V600E^ inhibitors such as vemurafenib, dabrafenib, and encorafenib and inhibitors of MEK, including trametinib, cobimetinib, and binimetinib, have markedly improved clinical outcomes for patients with BRAF-mutant melanoma. Nevertheless, drug resistance and melanoma recurrence often follow the initial response to treatment [2,3]. Therefore, despite advances in treatment options, melanoma remains a persistent challenge and novel molecular targets to combat drug resistance are needed. Resistance to targeted therapy arises from MAPK pathway reactivation, increased activity of alternative signaling pathways, and overexpression of pro-survival proteins, such as survivin, a member of inhibitor of apoptosis (IAP) proteins [4,5,6]. Survivin, also known as baculoviral inhibitor of apoptosis repeat-containing 5 (BIRC5), has attracted significant attention as a potential target in anticancer therapy [7], primarily due to its distinct expression in normal and neoplastic cells [7,8]. Survivin exerts an important role in embryogenesis, whereas in normal cells its expression is very low or undetectable. By contrast, high expression of survivin is often detected in a wide variety of cancers, such as breast cancer [9], lung cancer [10], glioblastoma multiforme [11], colorectal cancer [12], and melanoma [13], thereby enabling cancer cell survival by reducing caspase activity and preventing apoptosis. Moreover, survivin overexpression has been associated with the development of chemotherapy resistance, cancer recurrence, and short-term survival [7,8,14]. Since its discovery in 1997 by Ambrosini et al. [15], numerous studies have provided valuable insights into the functional role of survivin, and multiple strategies for targeting this protein have been proposed. These approaches include: (a) molecules targeting the transcription of survivin; (b) molecules targeting survivin translation; (c) molecules targeting the binding site of Smac/Diablo within survivin; (d) molecules targeting the survivin dimerization interface; (e) molecules targeting heat shock protein-90 (HSP-90), the interacting partner of survivin; and (f) vaccines and immunotherapeutic approaches against survivin. Several compounds have advanced through clinical trials across different cancer types, including melanoma [7,14]; however, the lack of identifiable active sites and enzymatic activity makes survivin difficult to target [16]. Therefore, indirect targeting of survivin is considered a therapeutic option. The expression of survivin is influenced by various oncogenic regulators and pathways that are often dysregulated in human malignancies, e.g., Notch [17,18], Yes-associated protein (YAP) [19], Hedgehog [20], epidermal growth factor receptor (EGFR) [21], phosphatidylinositol 3-kinase/protein kinase B (PI3K/Akt) [22,23], MAPK [24,25], NF-κB [26], mTOR [27], signal transducer and activator of transcription 3 (STAT3) [28], and CREB-binding protein (CBP)/β-catenin [29,30]. Inhibiting the CBP/β-catenin axis offers a potential benefit for treating patients with various types of cancer, including melanoma, as it modulates the activity of β-catenin instead of blocking the entire pathway [31].

One such compound, PRI-724, binds with high affinity to the N-terminus of CBP and influences the expression of β-catenin target genes associated with cancer cell survival (e.g., survivin, c-Myc, and cyclin D1) while leaving p300-mediated β-catenin activity unaffected [31]. The antiproliferative activity of PRI-724 in various types of cancer, including testicular germ cell tumors (GCTs) [32], neuroendocrine tumors [33], head and neck carcinoma [34], osteosarcoma [35], hepatocellular carcinoma [36,37], soft tissue sarcomas [38], lung adenocarcinoma [39] and acute and chronic myeloid leukemia [40,41], has been already investigated; however, to the best of our knowledge, PRI-724 activity has not been evaluated in melanoma so far. The inhibitory activity of PRI-724 has been evaluated in clinical trials for cancers such as pancreatic cancer (NCT01764477; phase I), leukemia (NCT01606579; phase I and II), and colorectal cancer (NCT01302405 phase I; NCT02413853; phase II), and other conditions, including primary biliary cholangitis patients (NCT04047160, phase I) and liver cirrhosis patients (NCT02195440, phase I; NCT03620474, phase I and phase 2; NCT02828254, observational study). Although several clinical trials have been completed, the results are still awaited, and PRI-724 has not yet been approved for use as an anticancer drug.

This study aimed to investigate the molecular effects of PRI-724 and their cellular consequences in patient-derived drug-naïve melanoma cells and their vemurafenib- and trametinib-resistant counterparts. The results presented herein shed new light on the activity of PRI-724 in melanoma cells and support its potential as a chemotherapeutic agent for the treatment of melanoma patients.

## 2. Materials and Methods

### 2.1. Compounds

Trametinib (GSK1120212; #S2673), vemurafenib (PLX4032; #S1267), and PRI-724 (#S8968) were purchased from Selleck Chemicals LLC (Houston, TX, USA) and used at 50 nM, 10 μM, and 0.075 μM–5 μM, respectively.

### 2.2. Generation and Culture of Drug-Naïve and Trametinib- and Vemurafenib-Resistant Melanoma Cells

**Figure 1:**
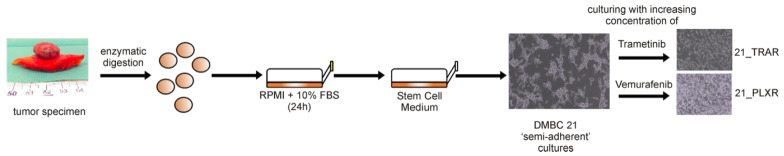
Simplified scheme of DMBC21 isolation and generation of trametinib-resistant (21_TRAR) and vemurafenib-resistant (21_PLXR) melanoma cells. The DMBC21 and 21_TRAR cell lines are classified as ‘semi-adherent’ cultures, consisting of both suspended and adherent cells. By contrast, 21_PLXR melanoma cells are representative of adherent cell cultures.

All experiments were conducted using patient-derived melanoma cell lines that were obtained during surgical interventions and extensively characterized [42,43,44]. The study received approval from the Bioethics Committee of the Medical University of Lodz (identification code: RNN/84/09/KE), and informed consent was obtained from the patient before the acquisition of tumor tissue. The drug-naïve DMBC21 cell line was derived from a melanoma specimen obtained from the back of a 65-year-old female patient. The melanoma was characterized as Clark level IV, with a Breslow thickness of 10 mm, mitotic index (MI) of 1, and classified as pIIC according to the seventh edition of the American Joint Committee on Cancer (AJCC) staging guidelines for melanoma. Melanoma cells were cultured in stem cell medium (SCM), as previously described [44]. Patient-derived melanoma cells were established cell lines with unlimited proliferative potential, necessary for long-term growth and generation of resistant cell lines [43,44]. To generate the vemurafenib-resistant cell line (21_PLXR) or trametinib-resistant cell line (21_TRAR), DMBC21 melanoma cells, harboring a heterozygous mutation leading to BRAF^V600E^ were grown in SCM in the presence of gradually increasing concentrations of the respective drugs for 4–5 months to achieve high viability at a final concentration 50 nM for trametinib and 10 μM for vemurafenib [43]. A schematic overview of melanoma cell isolation and derivation of the trametinib- and vemurafenib-resistant counterparts is presented in Figure 1.

### 2.3. Assessment of Acid Phosphatase Activity (APA)

To quantify viable cells, acid phosphatase activity was evaluated via colorimetric analysis. Briefly, DMBC21, 21_TRAR, and 21_PLXR melanoma cells were seeded at a density of 3.2 × 10^3^ per well in 96-well plates. Cells were exposed to PRI-724 for 24, 48, and 72 h at 37 °C and the enzymatic assay was conducted, as previously described [43].

### 2.4. Time-Lapse Microscopy (IncuCyte^®^ ZOOM)

A time-lapse fluorescence microscope system, IncuCyte^®^ ZOOM (Essen Bioscience, Essen, Germany), was used to monitor the real-time changes in cell confluence during exposure to PRI-724. Specifically, DMBC21, 21_TRAR, and 21_PLXR melanoma cells at a density of 7 × 10^3^ per well in 96-well plates were exposed to PRI-724 and monitored every 4 h for up to 72 h. The area occupied by cells was quantified over time as the percentage of cell confluence. Data analysis was conducted using IncuCyte^®^ ZOOM software v2015A.

### 2.5. Caspase-3/7 Activation Assessed by Time-Lapse Fluorescence Microscopy

DMBC21, 21_TRAR, and 21_PLXR melanoma cells at a density of 7 × 10^3^ per well in 96-well plates were exposed to PRI-724 and IncuCyte™ Caspase-3/7 Apoptosis Assay Reagent (#4440, Sartorius, Göttingen, Germany) at 4 μM. The activation of caspase-3/7 was evaluated at four-hour intervals utilizing a time-lapse fluorescence microscope system (IncuCyte^®^, Essen Bioscience, Essen, Germany) over a period of 72 h. Image quantification was conducted using the IncuCyte^®^ ZOOM basic analyzer (Essen Bioscience, Essen, Germany). The percentage of cells exhibiting active caspase-3/7 was determined by calculating the proportion of confluent apoptotic cells relative to the total confluence.

### 2.6. Flow Cytometry

DMBC21, 21_TRAR, and 21_PLXR melanoma cells were seeded at a density of 75 × 10^3^ per well in 24-well plates and incubated with PRI-724 for up to 48 h. After double-staining with an apoptosis detection kit consisting of propidium iodide (PI) and FITC-conjugated Annexin V (BD Biosciences, San Jose, CA, USA), samples were assessed using a FACSVerse flow cytometer (BD Biosciences, San Jose, CA, USA) and analyzed using BD FACSuite software v1.0.5.3824.

### 2.7. Western Blotting

DMBC21, 21_TRAR, and 21_PLXR melanoma cells were seeded at density of 4 × 10^5^ in 6-well plates and incubated with PRI-724 at the concentration range of 0.15–0.63 μM for 48 h. Cell lysates were prepared as described elsewhere [43] and stored at −80 °C until analysis. Samples with a total of 15 μg of protein were then loaded onto a 7% or 12% SDS-polyacrylamide gel, followed by electrophoresis conducted at a constant voltage of 25 V/cm. Next, western blotting was conducted as reported earlier [43]. Primary antibodies detecting survivin (#2808), p21 (#2947), cyclin D1 (#92G2), c-Myc (#E5Q6W), and MITF (#12590) were obtained from Cell Signaling Technology (Danvers, MA, USA). GAPDH (#SC-47724, Santa Cruz Biotechnology, Santa Cruz, CA, USA) and β-actin (#13E5, Cell Signaling Technology, Danvers, MA, USA) were used as loading controls. Secondary antibodies, specifically horseradish peroxidase (HRP)-conjugated anti-mouse (#7076S) and anti-rabbit antibodies (#7074), were obtained from Cell Signaling Technology (Danvers, MA, USA). Membranes were treated with Pierce^®^ ECL Western Blotting Substrate (Pierce, Rockford, IL, USA) for one minute and subsequently visualized utilizing the ChemiDoc Imaging System (Bio-Rad, Hercules, CA, USA). ImageJ software v1.54p was used to conduct densitometric analysis.

### 2.8. Invasion Assays

Corning^®^ BioCoat^®^ Matrigel^®^ Invasion Chambers with 8.0 µm polyethylene terephthalate (PET) membranes (#4480, Corning, Corning, NY, USA) were used to assess cell invasion. DMBC21, 21_TRAR, and 21_PLXR melanoma cells (2.5 × 10^4^) were exposed to PRI-724 at 0.3 μM and 0.63 μM and kept in the Matrigel Invasion Chambers for 48 h at 37 °C. A solution of 10% FBS was used as the chemoattractant. Non-invading cells at the upper surface of the membrane were removed using a cotton-tipped swab, whereas the cells adhering to the lower surface were fixed with methanol, stained with hematoxylin and eosin, and subsequently microphotographed (CKX53 microscope, Olympus, Tokyo, Japan) and quantified. The invasive potential of DMBC21, 21_TRAR, and 21_PLXR melanoma cells was additionally assessed using the IncuCyte^®^ Scratch Wound Assay (IncuCyte^®^, Essen Bioscience, Essen, Germany). DMBC21, 21_TRAR, and 21_PLXR melanoma cells plated at a density of 10^5^ per well in IncuCyte^®^ Imagelock 96-Well Plates (#BA-04857, Sartorius, Göttingen, Germany), previously coated with 100 mg/mL Matrigel^®^ (50 μL/well) (#354234, Corning, Corning, NY, USA), were incubated for 24 h at 37 °C to ensure cell attachment. Next, the confluent cell monolayer was wounded using the 96-well IncuCyte^®^ 96-Well Woundmaker Tool (#4563, Sartorius, Göttingen, Germany). After wounding, the medium was removed and wells were washed twice with DMEM F-12. Next, DMBC21, 21_TRAR, and 21_PLXR melanoma cells were carefully overlaid with 50 μL of the Matrigel^®^ top layer (6 mg/mL) supplemented with PRI-724 at 0.3 μM and 0.63 μM. After polymerization (30 min, 37 °C), 100 μL of the top medium supplemented with PRI-724 at 0.3 μM and 0.63 μM was added. Mitomycin C (MMC) at 1.5 μg/mL was used to exclude the effect of proliferation. The plates were scanned every 2 h up to 48 h using the IncuCyte^®^ Live-Cell Analysis System. Relative wound density was estimated using IncuCyte^®^ ZOOM software v2015A.

### 2.9. RNA Sequencing Analysis

RNA was isolated from drug-naïve DMBC21 cells and their drug-resistant counterparts, 21_TRAR and 21_PLXR cells, using the miRNeasy Mini Kit (#217004, Qiagen, Hilden, Germany), according to the manufacturer’s protocol. Briefly, cells were incubated in lysis buffer, and the lysates were passed through spin columns to bind RNA. RNA was eluted in RNase-free water and stored at −80 °C. RNA concentration and purity were assessed using a NanoDrop 2000 spectrophotometer (Thermo Fisher Scientific, Waltham, MA, USA) and RNA integrity was evaluated using a 2100 Bioanalyzer (Agilent Technologies, Santa Clara, CA, USA). Only samples that passed the purity and integrity thresholds were used for library preparation. The Ribo-Zero rRNA Removal Kit (Epicentre, Madison, WI, USA) was used to deplete rRNA. The NEBNext^®^ Ultra^TM^ Directional RNA Library Prep Kit for Illumina^®^ (New England Biolabs, Ipswich, MA, USA) was used to generate sequencing libraries, and unique index codes were added to each sample. Briefly, NEBNext First Strand Synthesis Reaction Buffer was used for the fragmentation of RNA at an elevated temperature. First-strand cDNA was synthesized using random primers and reverse transcriptase. Second-strand cDNA was synthesized using DNA Polymerase I and RNase H. The remaining overhangs were converted into blunt ends. After adenylation of the 3′ ends of DNA fragments, NEBNext Adaptor was ligated. To select inserts of 150~200 bp, the library fragments were purified with AMPure XP Beads (Beckman Coulter, Beverly, MA, USA). Then, 3 μL of USER Enzyme (New England Biolabs, Ipswich, MA, USA) was used with size-selected, adaptor-ligated cDNA at 37 °C for 15 min. PCR was then performed with Phusion High-Fidelity DNA polymerase, Universal PCR primers, and Index(X) Primer. PCR products were purified, and library quality was evaluated using an Agilent Bioanalyzer 2100 and qPCR. Sequencing was performed on the Illumina NovaSeq 6000 (Illumina, San Diego, CA, USA) platform to generate 150 bp paired-end reads with an average read depth of 50 M reads per sample. The HISAT2 (v2.0.4) tool was used to map the RNA-seq reads and to obtain information on read location on the reference genome (*Homo sapiens* GRCh38.113.HW). StringTie (v2.1.4) was used to assemble the mapped reads and calculate the FPKMs of coding genes in each sample. Raw and processed RNA-seq data submitted to Gene Expression Omnibus (GEO) database are freely available under accession number GSE301849.

### 2.10. Bioinformatics Analysis

The SKCM (skin cutaneous melanoma) dataset from The Cancer Genome Atlas (TCGA), containing transcriptional and clinical data from 461 melanoma patients, was used to evaluate the expression of *BIRC5* and its impact on melanoma patient survival. The Gene Expression Profiling Interactive Analysis 2 (GEPIA2) online tool (http://gepia2.cancer-pku.cn/ (accessed on 30 June 2025)), which integrates TCGA data with Genotype-Tissue Expression (GTEx) datasets of normal tissues, was used to assess differential expression of *BIRC5* between melanoma and normal tissues. The Prediction of Clinical Outcomes from Genomic Profiles (PreCOG) online tool (https://precog.stanford.edu/ (accessed on 30 June 2025)) was used to correlate *BIRC5* expression with overall survival of melanoma patients. Kaplan–Meier survival curves were generated to visualize survival differences between patients with high and low *BIRC5* expression, and the log-rank test was used to check for statistical significance. The TCGA mutation oncoplot was prepared using the maftools Bioconductor package (v2.18.0), and a multivariate Cox regression analysis model for the forest plot was evaluated using the survivalROC (v1.0.3.1) and survminer (v0.5.0) packages in R (v4.4.2). Downloading and merging TCGA SKCM transcriptional and clinical data were performed using the TCGAbiolinks package (v2.30.4) in R, and the forestplot was created using the forestplot unction. The GSE50509 and GSE95704 microarray datasets from the Gene Expression Omnibus (GEO) were reanalyzed in R. Differentially expressed genes were identified using the limma Bioconductor package (v3.58.1), applying thresholds of |Fold Change| ≥ 1.5 and Benjamini–Hochberg adjusted *p*-value ≤ 0.05. The volcano plot and heatmap of differentially expressed genes (DEGs) were generated using the pathlinkR (v1.6.0) and pheatmap (v1.0.12) packages in R, respectively. Gene ontology (GO) and Kyoto Encyclopedia of Genes and Genomes (KEGG) pathway enrichment analyses were performed using the clusterProfiler library (v4.18.0) and visualized using the ggplot2 graphical library (v3.5.1) in R. All statistical calculations regarding bioinformatics analyses were conducted within the R environment (v4.4.2).

### 2.11. Statistical Analysis

All statistical calculations were conducted using GraphPad Prism 9 software v 9.0.0. To determine significant differences between the mean values of the tested parameters, the Student’s *t*-test was used. Differences were considered statistically significant at *p* < 0.05.

## 3. Results

### 3.1. PRI-724 Reduces Viability of Drug-Naïve and Trametinib- and Vemurafenib-Resistant Melanoma Cells

**Figure 2:**
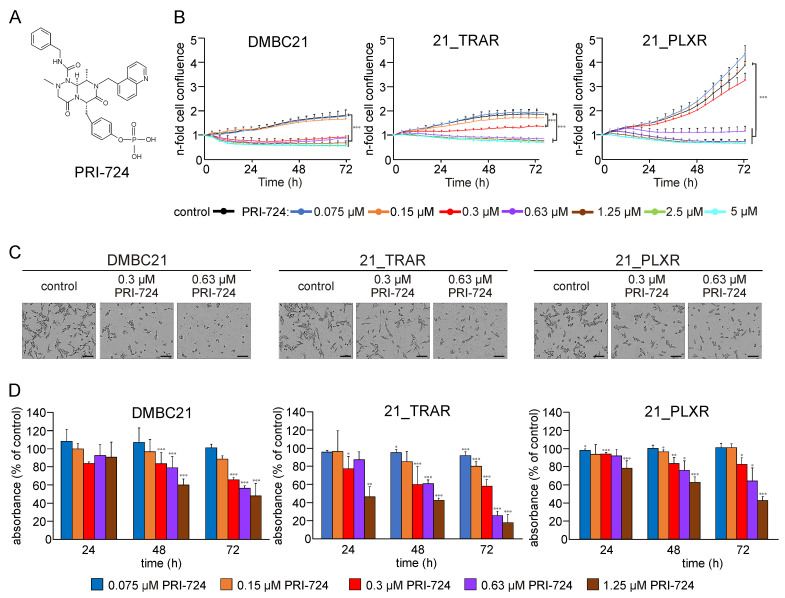
PRI-724 exhibits cytotoxic effects in melanoma cells. (**A**) Chemical formula of PRI-724. (**B**) Proliferation time-course curves for PRI-724-treated DMBC21, 21_TRAR, and 21_PLXR melanoma cells. Changes in the occupied area (% confluence) over time are shown as fold-change relative to the confluence at time T_0_. Plots represent mean values ± SD from a representative experiment conducted in triplicate. (**C**) Microphotographs of DMBC21, 21_TRAR, and 21_PLXR cells exposed to PRI-724 at 0.3 and 0.63 μM for 48 h. Scale bar, 100 μm. (**D**) Percentage of cell viability relative to the control for up to 72 h of PRI-724 treatment based on the activity of acid phosphatase. Data are presented as mean ± SD; *n* = 3. Statistical significance (* *p* < 0.05; ** *p* < 0.01; *** *p* < 0.001) was evaluated relative to respective untreated controls.

To determine the potential of targeting the CBP/β-catenin interaction, we tested the antitumor effects of PRI-724 (Figure 2A) against drug-naïve and trametinib- and vemurafenib-resistant melanoma cells. First, the effective concentrations of PRI-724 were determined in DMBC21, 21_TRAR, and 21_PLXR cells. To assess changes in cell proliferation and morphology in real time, time-lapse microscopy was performed using the IncuCyte^®^ ZOOM system (v2015A). It was observed that PRI-724 inhibited melanoma cell confluence in both concentration- and time-dependent manners. The most pronounced effects in reducing cell confluence were detected at concentrations of 0.63 μM or higher, which completely restrained an increase in cell confluence (Figure 2B). By contrast, lower concentrations of 0.075 μM and 0.15 μM exhibited a minimal impact, while a concentration of 0.3 μM resulted in a moderate reduction in confluence, particularly in DMBC21 and 21_TRAR cells. With the increase in PRI-724 concentration, notable morphological alterations marked by cell rounding and loss of adherence were observed after 48 h of drug treatment (Figure 2C). Notably, DMBC21 and 21_TRAR cells started to lose cell adherence at 0.3 μM, whereas 21_PLXR cells manifested similar changes at a higher concentration of PRI-724 (0.63 μM). Additionally, time-course changes (24–72 h) in the morphology of DMBC21, 21_TRAR, and 21_PLXR cells exposed to PRI-724 at 0.3 and 0.63 μM are presented in microphotographs in Supplementary Figure S1.

To further evaluate the effect of PRI-724 on the viability of melanoma cells, the APA assay, quantifying the metabolic activity of cells, was used. PRI-724 reduced the viability of DMBC21, 21_TRAR, and 21_PLXR melanoma cells in concentration- and time-dependent manners (Figure 2D). Moreover, flow cytometry was utilized to identify PI-positive cells following PRI-724 treatment (Supplementary Figure S2). The presented results highlight the significant cytotoxic efficacy of PRI-724 in DMBC21, 21_TRAR, and 21_PLXR cells and indicate a response that is dependent on both time and concentration, with trametinib-resistant cells demonstrating the highest susceptibility to PRI-724.

### 3.2. PRI-724 Effectively Induces Apoptosis in a Concentration-Dependent Manner in Drug-Naïve and Trametinib- and Vemurafenib-Resistant Melanoma Cells

**Figure 3:**
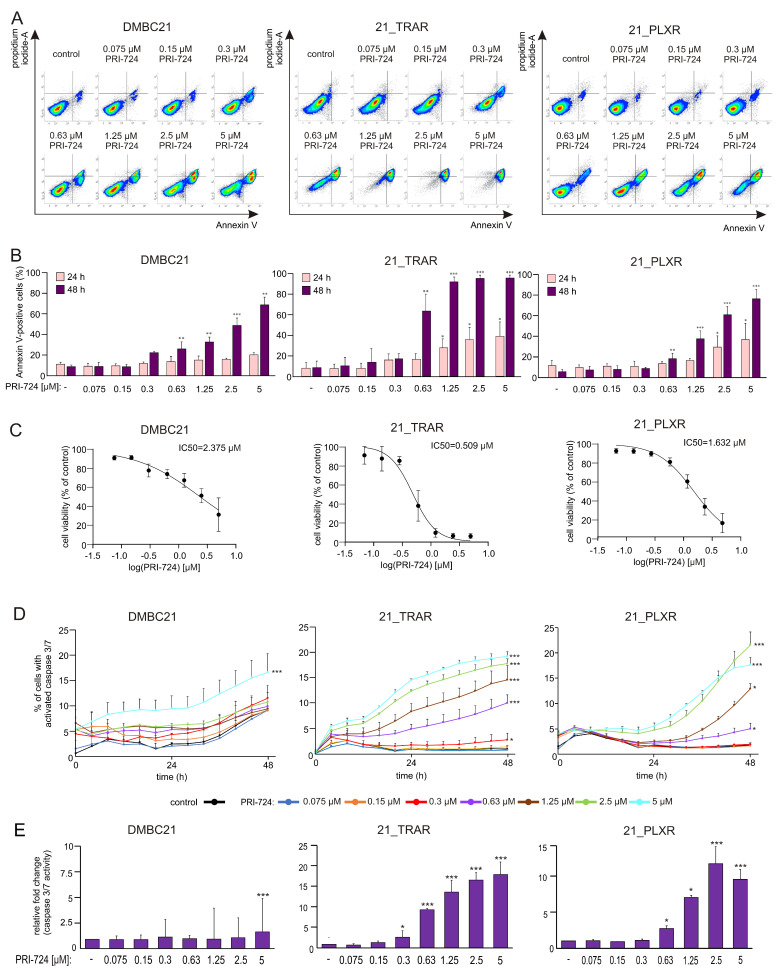
PRI-724 induces apoptosis in drug-naïve (DMBC21) and trametinib- (TRAR-) and vemurafenib- (PLXR-) resistant melanoma cells. (**A**) Representative flow cytometry dot plots of DMBC21, 21_TRAR, and 21_PLXR melanoma cells stained with Annexin V/propidium iodide (PI) after 48 h of incubation with PRI-724 at indicated concentrations. (**B**) Percentages of Annexin V-positive cells (early apoptotic cells (Annexin V^+^/PI^−^) and late apoptotic cells (Annexin V^+^/PI^+^)) after PRI-724 treatment for 24 and 48 h assessed by flow cytometry. Data are presented as mean ± SD; *n* = 3. (**C**) Dose–response curves exhibiting the percentage of viable cells (Annexin V^−^/PI^−^) exposed to PRI-724 for 48 h compared to control cells. IC_50_ values are indicated. *n* = 3. (**D**) Percentages of apoptotic DMBC21, 21_TRAR, and 21_PLXR melanoma cells with high caspase 3/7 activity after PRI-724 treatment over the course of 48 h were assessed using the time-lapse imaging system, IncuCyte^®^ ZOOM. Results of a representative experiment performed in triplicate (±SD) are shown. (**E**) Caspase 3/7 activity represented as fold change relative to control after 48 h of PRI-724 treatment. Statistical significance (* *p* < 0.05; ** *p* < 0.01; *** *p* < 0.001) was evaluated relative to respective untreated controls.

The apoptosis-inducing activity of PRI-724 was detected as increased Annexin V positivity and caspase-3/7 activation. Double Annexin V/propidium iodide staining revealed that 21_TRAR cells were the most susceptible to PRI-724-induced apoptosis (Figure 3A,B). Significantly increased percentages of Annexin V-positive cells were already detected in trametinib- and vemurafenib-resistant cells after 24 h of treatment with PRI-724 at 1.25 μM and 2.5 μM, respectively. After 48 h of treatment with 0.63 μM PRI-724, 63% of 21_TRAR cells were Annexin V positive, indicating a pronounced induction of apoptosis. By contrast, DMBC21 and 21_PLXR melanoma cells exhibited lower sensitivity to PRI-724, with only 26% and 19% of apoptotic cells, respectively, under the same conditions. IC_50_ values were calculated from dose–response curves exhibiting the percentages of viable cells (Annexin V^−^/PI^−^) in drug-treated cells compared to control cells using the ‘log [inhibitor] vs. normalized response’ model. The IC_50_ values were 2.375 μM, 0.509 μM, and 1.632 μM for DMBC21, 21_TRAR, and 21_PLXR melanoma cells, respectively, after 48 h of treatment (Figure 3C). Moreover, time-lapse fluorescence microscopy (Figure 3D,E) revealed a substantial increase in the percentages of caspase-3/7-positive cells after PRI-724 treatment across all tested cell lines.

### 3.3. Treatment with PRI-724 Attenuates the Invasive Potential of Drug-Naïve and Trametinib-and Vemurafenib-Resistant Melanoma Cells

**Figure 4:**
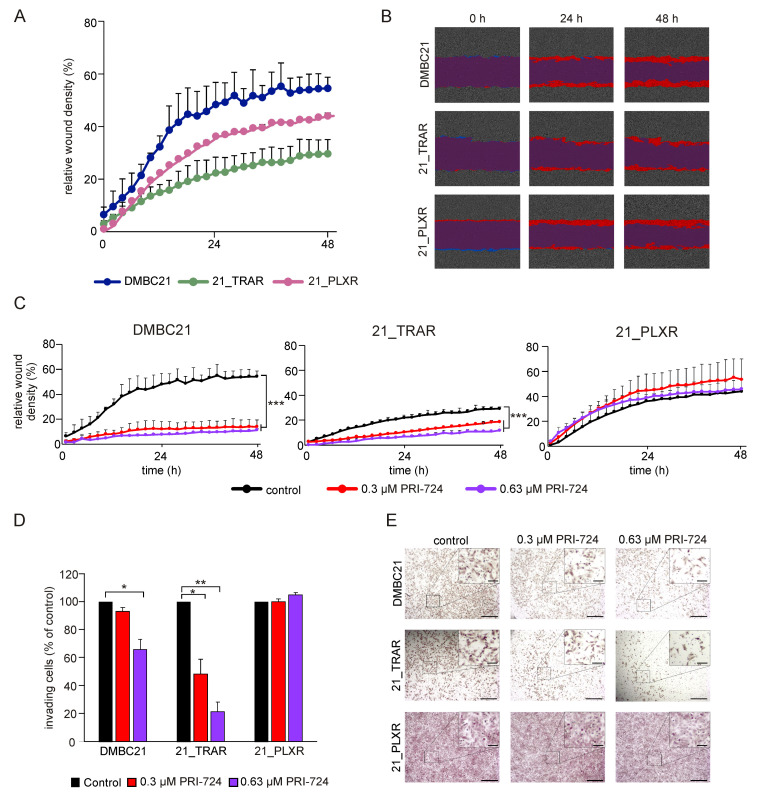
PRI-724 modulates the invasive potential of melanoma cells. (**A**) Relative wound density (RWD), defined as the ratio of the cell density in the wound over the cell density outside the wound, was measured every two hours for up to 48 h in DMBC21, 21_TRAR, and 21_PLXR cells. MMC at 1.5 µg/mL was used to exclude the effect of proliferation. (**B**) Representative phase-contrast images from the IncuCyte^®^ system showing the time-dependent increase of cell confluence in the wound area (red) for DMBC21, 21_TRAR, and 21_PLXR cells taken at 0 h, 24 h, and 48 h post-wounding. (**C**) Inhibitory effect of PRI-724 in a scratch-wound healing assay followed using the IncuCyte^®^ system for up to 48 h. MMC at 1.5 µg/mL was used to exclude the effect of proliferation. (**D**) Bar graph shows the percentages of invading cells after incubation with PRI-724 at the indicated concentrations for 48 h. Data are expressed as mean ± SD (*n* = 2). (**E**) Representative photomicrographs taken after incubation with PRI-724 at the indicated concentration for 48 h. Scale bar: 500 µm (main figure); 100 µm (magnified panel). Statistical significance (* *p* < 0.05; ** *p* < 0.01; *** *p* < 0.001) was evaluated relative to respective untreated controls.

Next, we examined the invasive potential of DMBC21, 21_TRAR, and 21_PLXR cells. Drug-naïve and trametinib- and vemurafenib-resistant melanoma cells were seeded in Matrigel-coated chambers and on Incucyte^®^ Imagelock 96-Well Plates to perform invasion and scratch wound invasion assays, respectively. Both methods revealed that 21_TRAR cells demonstrated lower invasiveness than DMBC21 and 21_PLXR cells (Figure 4A,B,E). When cells were treated with 0.3 μM or 0.63 μM PRI-724 and monitored for 48 h, a concentration-dependent decrease in invasive capacity was detected in the DMBC21 and 21_TRAR cell lines. It is noteworthy that PRI-724 significantly reduced 21_TRAR cell invasion to approximately 48% and 21% of the control at concentrations of 0.3 μM and 0.63 μM, respectively (Figure 4D). Conversely, PRI-724 had no measurable effect on the invasive capacity of vemurafenib-resistant cells (Figure 4C–E). Collectively, these findings suggested that inhibition of the CBP/β-catenin signaling axis by PRI-724 may suppress the invasive capacity of melanoma cells, with the most pronounced effect observed in trametinib-resistant cells.

### 3.4. The CBP/β-Catenin Signaling Pathway May Play a Significant Role in Development and Maintenance of Drug Resistance in Melanoma

**Figure 5:**
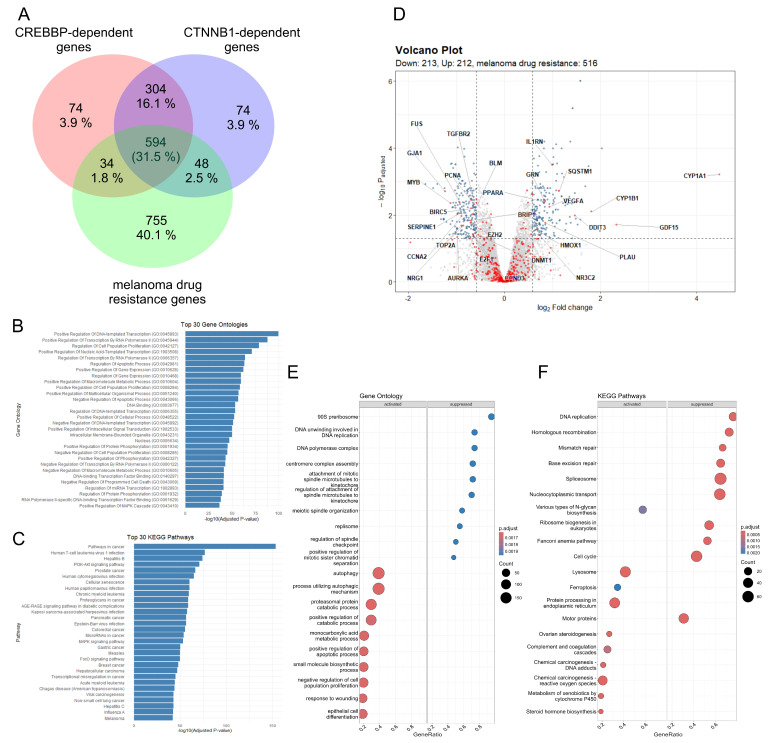
The CBP/β-catenin pathway is involved in melanoma drug resistance. (**A**) Venn diagram illustrating the intersection of genes associated with ‘*CREBBP*’, ‘*CTNNB1*′, and ‘melanoma drug resistance’, as identified by the GeneCards database. The intersection includes 594 common genes, representing more than 30% of all genes involved in melanoma drug resistance. A list of 594 genes was underwent over-representation analysis using GO categories (**B**) and KEGG pathways (**C**) to identify enriched biological processes and pathways. The top 30 over-represented GO and KEGG terms were illustrated using the clusterProfiler package in R, respectively. (**D**) The GSE95704 GEO dataset was reanalyzed to find differentially expressed genes in oral squamous cell carcinoma cell lines treated with ICG-001, applying thresholds of |Fold Change| ≥ 1.5 and Benjamini–Hochberg adjusted *p*-value ≤ 0.05 (blue dots). The volcano plot was generated using the pathlinkR package in R. Red dots represent previously identified 594 genes linked to the CBP/β-catenin pathway and melanoma drug resistance. Grey dots represent statistically insignificant genes. GO (**E**) and KEGG (**F**) enrichment analyses were performed on the differentially expressed genes identified in GSE95704. The top 10 activated and suppressed annotations were visualized using the clusterProfiler package in R, with an adjusted *p*-value cutoff of 0.05. Activated and suppressed annotations were identified according to positive and negative values of normalized enrichment score (NES), respectively. GeneRatio represents the number of input genes in the specific KEGG or GO term, divided by total number of input genes.

To explore the potential association between CBP/β-catenin signaling and melanoma drug resistance, the GeneCards website (https://www.genecards.org/) was used to extract gene lists related to the keywords ‘CREBBP-dependent genes’, ‘CTNNB1-dependent genes’ and ‘melanoma drug resistance genes’, giving 1006, 1020, and 1431 genes, respectively (relevance score ≥ 10.00). The intersection of all three gene lists yielded 594 genes common to all three terms (Figure 5A). This finding suggested that more than 30% of genes related to drug resistance in melanoma may be dependent on CBP/β-catenin signaling. Subsequently, over-representation analysis (ORA) was performed on the list of 594 common genes using the clusterProfiler package in R for GO terms (Figure 5B), which included Biological Process (BP), Cellular Component (CC), and Molecular Function (MF) annotations, and KEGG pathways (Figure 5C). Functional enrichment analysis of the set of 594 common genes revealed that pathways related to regulation of DNA replication and DNA repair, positive regulation of gene expression, and the cell cycle were over-represented, among others.

To predict possible molecular consequences of inhibiting the formation of the CBP/β-catenin complex in solid tumors, the GSE95704 dataset of head and neck cancer, derived from GEO, was reanalyzed [45]. This tumor is characterized by dysregulation of the CBP/β-catenin pathway, and its abnormal activity has been linked to the aggressiveness of head and neck cancer [45]. Raw transcriptomic data obtained after microarray signal processing of RNA samples from ICG-001-treated and control untreated oral squamous cell carcinoma (OSCC) cells were subjected to differential gene expression analysis using the limma Bioconductor package in R. ICG-001, a homolog of PRI-724, specifically inhibits CBP/β-catenin-dependent transcription. As a result, 425 genes were found to be differentially expressed between ICG-001-treated OSCC cell lines when compared with control samples. The volcano plot (Figure 5D) revealed that 212 genes were upregulated and 213 genes were downregulated after ICG-001 treatment. The spots highlighted in red represent a subset of 594 genes identified previously as CBP/β-catenin-dependent melanoma drug-resistance genes (Figure 5A). Among downregulated genes after ICG-001 treatment, *BIRC5*, *EZH2*, *E2F1*, and other oncogenes related to melanoma were found (Supplementary Tables S2 and S3). Subsequently, a list of 425 differentially expressed genes, along with their log_2_ fold change values, was assessed using the clusterProfiler package in R to find activated and suppressed GO (Figure 5E) and KEGG (Figure 5F) pathways for OSCC cells treated with ICG-001 compared with untreated cells. Suppressed genes associated with DNA replication and repair and the cell cycle, along with activated genes involved in various types of cell death, were identified. These findings further suggested that, as in head and neck cancer, inhibition of CBP/β-catenin signaling by small-molecule inhibitors ICG-001 or PRI-724 may exert antiproliferative and proapoptotic effects in melanoma.

### 3.5. PRI-724 Downregulates Survivin (BIRC5)

**Figure 6:**
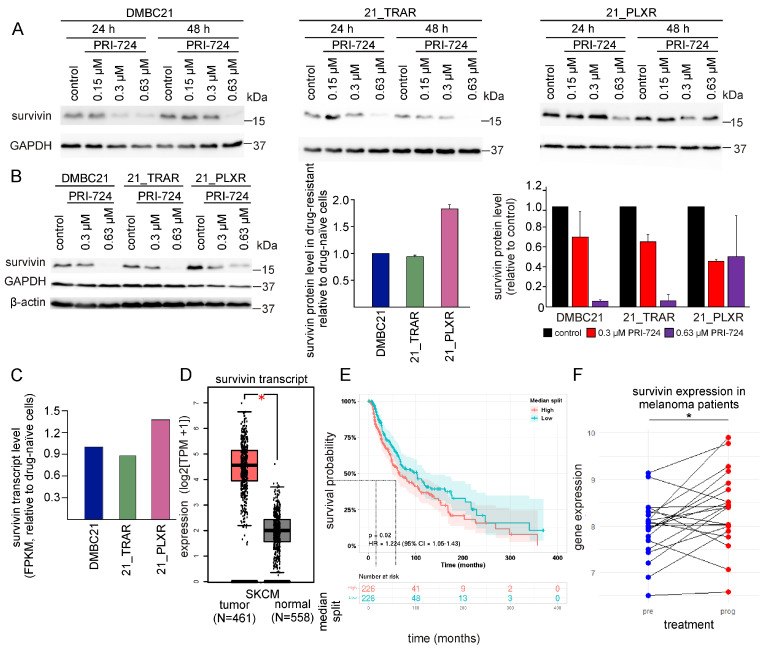
PRI-724 downregulates survivin (BIRC5), a predictor of poor prognosis and treatment response in melanoma. (**A**) Representative immunoblots showing levels of survivin in drug-naïve and trametinib- and vemurafenib-resistant melanoma cells treated with PRI-724 for 24 h and 48 h. GAPDH was used as a loading control. (**B**) Representative immunoblot comparing survivin protein levels in drug-naïve and trametinib- and vemurafenib-resistant melanoma cells along with its levels after treatment with PRI-724 (48 h). Densitometric analysis of survivin expression in trametinib- and vemurafenib-resistant melanoma cells relative to drug-naïve cells, followed by quantitative assessment of survivin protein levels after treatment with PRI-724 (48 h), assessed against respective controls and normalized to the β-actin protein level. ImageJ software was used to quantify protein band intensities. Data are presented as mean ± SD from two independent experiments. (**C**) RNA-seq data comparing the expression of survivin in trametinib- and vemurafenib-resistant cell lines compared with drug-naïve cells. Relative expression levels were calculated based on FPKM values. (**D**) Elevated expression of survivin was observed in the SKCM TCGA dataset comparing 461 melanoma patient samples with 558 normal tissues from the GTEx database. Data are visualized using a boxplot prepared using the GEPIA2 tool. * *p*-value < 0.05. (**E**) Kaplan–Meier curve of overall survival according to survivin expression in melanoma patients, stratified by its median expression from the SKCM TCGA dataset. The *p*-value, hazard ratio (HR), and 95% confidence interval (CI) are indicated. The log-rank test was calculated, and the Kaplan–Meier plot was generated using the PreCOG tool. (**F**) Paired patient expression analysis of *BIRC5* before BRAFi treatment (pre) and during disease progression after treatment (prog). GEO dataset used: GSE50509; * *p*-value < 0.05.

Bioinformatics analysis revealed that *BIRC5*, which encodes survivin, was one of the genes downregulated after the inhibition of CBP/β-catenin signaling in OSCC cells (Figure 5D, Supplementary Table S3). Taking into consideration its significant role in melanoma development and drug resistance [5,6,13], we sought to determine whether PRI-724 could indirectly modulate survivin expression. The results of western blotting showed a dose-dependent reduction of survivin protein levels in DMBC21, 21_TRAR, and 21_PLXR cells at two time-points (24 h, 48 h) (Figure 6A). Quantitative data comparing survivin expression in drug-naïve and drug-resistant melanoma cells and PRI-724 effects obtained after 48 h of incubation are shown in Figure 6B. The results indicated that vemurafenib-resistant melanoma cells exhibited the highest expression of survivin among untreated cells, and these cells were the least responsive to PRI-724 at 0.63 μM. The obtained results were in accordance with the RNA-seq data, where the survivin transcript level was approximately 1.5-fold higher in 21_PLXR cells compared with both drug-naïve and trametinib-resistant cells (Figure 6C and  Figure S3). To find out whether our results were supported by clinical data, the SKCM dataset from TCGA containing mRNA expression data and clinical information gathered from 461 melanoma patients [46] was reanalyzed in terms of *BIRC5* expression and its prognostic value. Since the SKCM dataset is devoid of transcriptional data from normal skin samples, *BIRC5* expression data from TCGA was merged with GTEx transcriptional data represented by 558 normal human skin tissues. Boxplots created with the GEPIA2 tool revealed that the expression of *BIRC5* was significantly increased in melanoma patients compared with normal control tissue samples (Figure 6D). Furthermore, increased expression of survivin was associated with poor overall survival of melanoma patients. To evaluate the prognostic role of the expression of *BIRC5*, transcriptomic data derived from the SKCM dataset was utilized to prepare Kaplan–Meier survival plots using the PreCOG tool. Melanoma patients were stratified according to the expression values of *BIRC5* into high (above median *BIRC5* expression) and low (below median *BIRC5* expression) groups. The comparison of Kaplan–Meier survival curves corresponding to patients with high *BIRC5* expression showed a median survival probability of approximately 69 months, whereas patients with low *BIRC5* expression had a median survival probability of approximately 107 months. These data suggested that high *BIRC5* expression is an indicator of poor prognosis in melanoma patients (Figure 6E). Additionally, the GEO dataset GSE50509 [47], which contains transcriptomic data for melanoma patients who experienced disease progression after treatment with BRAF^V600^ inhibitors (prog) matched with pre-treatment melanoma samples (pre), was analyzed to assess changes in *BIRC5* expression. Increased *BIRC5* expression in patients that progressed on BRAF^V600^ inhibitor treatment became apparent, as the mean value in the pre-treatment group was 7.94 compared with 8.36 in the progression group (*p* = 0.02) (Figure 6F). This was in agreement with our immunoblotting results (Figure 6B). All of these results highlighted the role of survivin in melanoma and suggested that survivin inhibition may improve treatment efficacy and outcomes for melanoma patients, including those resistant to targeted therapy.

### 3.6. PRI-724 Downregulates the Level of CBP/β-Catenin Dependent Proteins in Drug-Naïve and Trametinib- and Vemurafenib-Resistant Melanoma Cells

**Figure 7:**
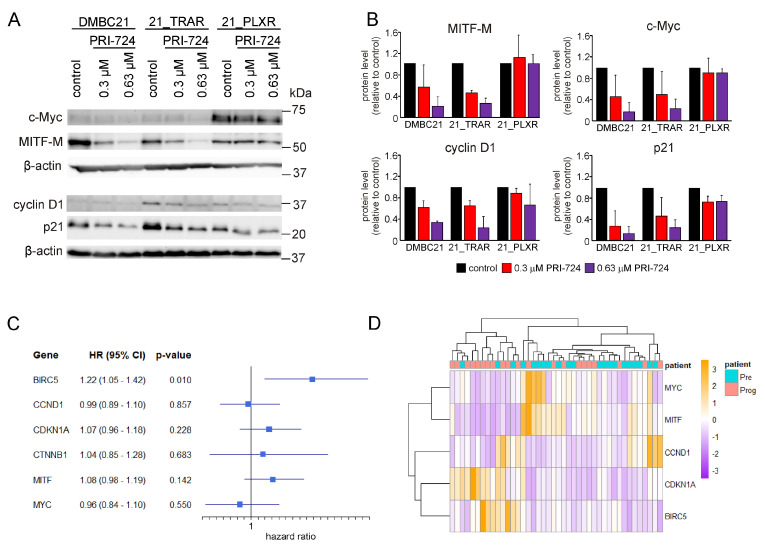
Pharmacological inhibition of the CBP/β-catenin axis decreases the expression of β-catenin-dependent genes and p21 level in drug-naïve and trametinib- and vemurafenib-resistant melanoma cells. (**A**) Representative immunoblots showing levels of c-Myc, cyclin D1, MITF-M, and p21 in drug-naïve and trametinib- and vemurafenib-resistant melanoma cells after 48 h of treatment with PRI-724. β-actin was used as a loading control. (**B**) Quantitative data indicating the fold change in protein levels of the specified targets relative to control, normalized to the β-actin protein level. Protein band intensities were quantified using ImageJ software. Data are presented as mean ± SD from two independent experiments. (**C**) A multivariable Cox proportional hazards model was used to evaluate the association between gene expression and overall survival in melanoma patients. Among several genes important for melanoma progression and drug resistance, only increased expression of *BIRC5* was identified as an independent statistically significant prognostic marker of poor survival. Hazard ratios (HR), 95% confidence intervals (CI), and *p*-values are shown for each gene included in the analysis. (**D**) Heatmap illustrating the expression of *BIRC5*, *CCND1*, *MITF*, *CDKN1A* (encoding p21), and *MYC*, genes widely associated with drug resistance in melanoma, using the GSE50509 dataset. This dataset contains transcriptomic data for melanoma patients who experienced disease progression following BRAFi treatment (prog), compared with their pre-treatment samples (pre). Differential expression levels are shown as z-scores, representing the relative expression of each gene across all samples.

To further investigate the effects of PRI-724 on the CBP/β-catenin signaling axis, we examined the expression of selected β-catenin-dependent genes encoding c-Myc, MITF-M, and cyclin D. PRI-724 reduced the levels of c-Myc, MITF-M, and cyclin D1 proteins in DMBC21, 21_TRAR, and 21_PLXR cells. Moreover, PRI-724 treatment efficiently downregulated p21 in a concentration-dependent manner in drug-naïve and trametinib- and vemurafenib-resistant melanoma cells (Figure 7A,B). Since PRI-724 significantly diminished the levels of c-Myc, MITF-M, cyclin D1, and p21, we were interested in whether downregulation of these genes may possess any prognostic value. To this end, the expression of *MYC*, *MITF*, *CCND1* (encoding cyclin D1), and *CDKN1A* (encoding p21), along with *BIRC5* and *CTNNB1* (encoding β-catenin), was merged with clinical data regarding survival time of melanoma patients from the TCGA SKCM dataset [46] using the TCGA biolinks workflow in R. The survival and survminer packages were then used to fit the multivariate Cox regression model (Figure 7C). Among the tested genes, only *BIRC5* expression in melanoma patients could be used as a statistically significant prognostic marker of poor outcome (HR 1.22; 95% CI: 1.05–1.42; *p*-value 0.01). Additionally, the GEO dataset GSE50509 [47], which contains transcriptomic data for melanoma patients who experienced disease progression after BRAFi treatment (prog) matched with pre-treatment melanoma samples (pre), was analyzed to assess the expression of genes associated with melanoma drug resistance and progression. The heatmap (Figure 7D) illustrated the distinct transcriptional profiles between pre-treatment and post-progression melanoma patient groups. Complete segregation of groups by hierarchical clustering was not fully achieved, likely due to high intrinsic melanoma heterogeneity and patient-related confounding factors such as age, gender, or ethnicity; therefore, interpretation of this heatmap and global transcriptional changes is challenging.

## 4. Discussion

Drug resistance remains a major clinical challenge, leading to melanoma recurrence and patient mortality. Identifying additional druggable targets is therefore essential for improving therapeutic outcomes and overcoming resistance to current treatments.

Our study focused on targeting the CBP/β-catenin interaction and provides evidence that inhibition of this signaling axis by PRI-724 modulates the invasive potential of melanoma cells and effectively induces apoptosis in time- and concentration-dependent manners. Fang et al. already observed that PRI-724 treatment significantly inhibited the migration and invasion of OS 143B osteosarcoma cells [35]. We found that the investigated melanoma cell lines displayed distinct invasive potentials, and their responses to PRI-724 treatment varied accordingly. PRI-724 reduced the invasive capacity of melanoma cells resistant to trametinib, a MEK inhibitor, whereas it had no significant effect on the invasiveness of cells resistant to vemurafenib, a BRAF^V600^ inhibitor. Correspondingly, melanoma cells resistant to the MEK inhibitor exhibited the highest caspase-3/7 activity following PRI-724 exposure. PRI-724 treatment has been shown to increase the levels of cleaved caspase-3/7, thereby promoting apoptosis in hepatocellular carcinoma cells [36]. Similarly, Schmidtova et al. [32] reported that apoptotic cell death in both drug-naïve and cisplatin-resistant testicular germ cell tumor cells was associated with elevated caspase-3/7 activity, with the highest caspase activation observed in cisplatin-resistant cells. He et al. [37] also reported that PRI-724 induced apoptosis in sorafenib-sensitive and -resistant hepatocellular carcinoma cells, with a markedly enhanced effect when combined with sorafenib. The results were further supported by in vivo studies employing a sorafenib-sensitive xenograft tumor model and consistent synergistic effects were confirmed in a 3D drug testing system with micro-dissected tumor tissues derived from these xenografts [37]. Correspondingly, PRI-724 induced apoptosis in chronic myeloid leukemia cells both in monotherapy and used alongside nilotinib [40]. Jiang et al. [41] also reported enhanced apoptosis in acute myeloid leukemia cells following a therapeutic regimen combining PRI-724 and sorafenib [41]. Moreover, both Zhou et al. and Jiang et al. [40,41] demonstrated the antitumor efficacy of PRI-724 in xenograft models, particularly when used in combination with tyrosine kinase inhibitors [40,41]. The effectiveness of dual therapy employing PRI-724 was also evaluated by Osawa et al., who indicated that PRI-724 combined with an anti-PD-L1 antibody resulted in tumor regression in a mouse model of colon cancer liver metastasis. This effect was associated with the accumulation of CD8^+^ T cells and phenotypic shift in macrophages toward a more cytotoxic state [48].

The molecular mechanisms underlying the activity of PRI-724 have been examined in various cancer types, including hepatocellular carcinoma [36,37], osteosarcoma [35], lung adenocarcinoma [39], soft tissue sarcomas [38], neuroendocrine tumors [33], head and neck squamous cell carcinoma [34], and acute and chronic myeloid leukemia [40,41]. PRI-724 treatment has been shown to downregulate survivin expression in several of these cancers, such as hepatocellular carcinoma [36,37], osteosarcoma [35], SARS-CoV-2-infected lung adenocarcinoma cells [39], and acute and chronic myeloid leukemia [40,41]. In the present study, we also demonstrated that PRI-724 downregulated survivin expression and other CBP/β-catenin-transcriptionally dependent proteins in patient-derived melanoma cells, especially those resistant to trametinib. To search for a possible explanation, we analyzed genomic and transcriptomic data. Whole-exome sequencing of DMBC21, 21_TRAR, and 21_PLXR cells revealed no mutations in the key components of the WNT/β-catenin signaling pathway; however, mutations present in 21_PLXR cells in key melanoma genes such as CDKN2A, DUSP6, ERBB2, or E2F3 might impact the resistance of vemurafenib-resistant cell lines to PRI-724 treatment (Supplementary Table S1). Transcriptomic analysis combined with immunoblotting revealed that survivin levels were higher in vemurafenib-resistant cells compared with their drug-naïve and trametinib-resistant counterparts. Elevated survivin expression may contribute to the diminished sensitivity of vemurafenib-resistant cells to PRI-724. These results are supported by those previously obtained showing that active β-catenin levels and phosphorylation of CREB at serine 133 (S133) are higher in vemurafenib-resistant cells in comparison with drug-naïve and trametinib-resistant cells [49]. Phosphorylation of CREB at S133 is a key activation step that enhances CREB-dependent transcription by promoting the recruitment of co-activators such as CBP [50], and transcriptional regulation of *BIRC5* is mediated by the CBP/β-catenin/TCF signaling axis [51,52,53,54,55,56,57]. Aberrant activation of this pathway has been identified as a key contributor to survivin overexpression in melanoma [58]. Bioinformatic analyses have indicated that survivin expression is significantly higher in melanoma than in normal skin, highlighting its potential role in oncogenic processes. Analysis of the GSE50509 dataset identified *BIRC5* among the genes upregulated in post-treatment samples taken from patients who exhibited disease progression following vemurafenib or dabrafenib therapy (Figure 6F). This suggests the role of survivin in adaptive resistance mechanisms and indicates that survivin may be a biomarker of poor prognosis [16]. Moreover, to gain a better understanding of the changes in individual gene expression of the CBP/β-catenin signaling axis in melanoma, an analysis of accessible datasets published in TCGA and GEO was performed. We found that the inhibition of the CBP/β-catenin axis mediated by ICG-001 in oral squamous cell carcinoma led to the downregulation of BIRC5, EZH2, AURKA, CCNA2, and other genes previously implicated in therapy resistance (Figure 5D). Furthermore, the results of functional enrichment analyses (GO and KEGG) suggested that ICG-001 not only modulates genes incorporated in proliferative and survival signaling pathways but also reactivates antitumor mechanisms, enhancing tumor sensitivity to therapeutic interventions (Figure 5E,F).

We further demonstrated that PRI-724 disrupted CBP/β-catenin signaling, leading to the downregulation of other key CBP/β-catenin dependent proteins, including cyclin D1, c-Myc, and MITF-M, in drug-naïve and trametinib- or vemurafenib-resistant cells. Our findings are consistent with those of other groups, as PRI-724 has been effective in downregulating CBP/β-catenin target proteins. Similarly, PRI-724 treatment resulted in downregulation of cyclin D1 in osteosarcoma cells, SARS-CoV-2-infected lung adenocarcinoma cells, and acute myeloid leukemia cells, as reported by Fang et al. [35], Kelch et al. [39], and Jiang et al. [41], respectively. Inhibition of CBP/β-catenin signaling also led to decreased cyclin D1 expression in soft tissue sarcomas [38] and neuroendocrine tumors [33]. Decreased cyclin D1 expression upon PRI-724 treatment resulted in significant reduction of the proliferative capacity of the above-mentioned cancer cells [33,35,38,39]. Consistent with our findings, Gabata et al. [36] and He et al. [37] reported that PRI-724 treatment resulted in reduced c-Myc expression in hepatocellular carcinoma cells. Similarly, inhibition of c-Myc was observed in chronic and acute myeloid leukemia cells following PRI-724 treatment, either as monotherapy or in combination with TKIs [40,41]. Moreover, PRI-724 upregulated p21 in hepatocellular carcinoma cells, leading to cell cycle arrest at the G0/G1 phase [36]. Cell cycle arrest in the G0/G1 phase was also observed in hepatocellular carcinoma cells co-administered sorafenib and PRI-724 [37]. Correspondingly, disruption of the β-catenin/CBP interaction by PRI-724 induced cell cycle deregulation in both uninfected and SARS-CoV-2-infected lung adenocarcinoma cells, suggesting that the effect was independent of viral infection [39], as well as in osteosarcoma cells [38]. Interestingly, PRI-724 decreased the p21 protein level in melanoma cells. p21 is widely recognized as a tumor suppressor and key transcriptional target of p53, mediating cell cycle arrest and DNA damage repair [59]. However, the function of p21 is highly context dependent and extends beyond its canonical role. In a p53-deficient environment, p21 may accumulate in the cytoplasm, where it acts as a pro-oncogenic factor, inhibiting apoptosis and promoting drug resistance [59,60,61]. The reduction in p21 level following CBP/β-catenin inhibition found in our study is in agreement with the previous findings reported for ICG-001 treatment in colon cancer cells [62].

Taken together, modulation of the CBP/β-catenin signaling pathway by PRI-724 may affect the expression of survivin and other genes implicated in carcinogenesis, tumor progression, and resistance to targeted therapies. Given the challenges associated with the direct targeting of survivin [14], we propose that targeting its upstream regulators, such as β-catenin, may represent a more effective and promising therapeutic strategy.

Selective inhibition of the CBP/β-catenin signaling axis represents a potentially safe approach, as selective modulation allows for the preservation of essential functions of canonical WNT signaling. This approach is advantageous as it maintains tissue homeostasis and facilitates repair processes without disrupting the entire β-catenin-dependent pathway [31]. However, despite the acceptable toxicity profile of PRI-724 [63,64,65], the lack of an oral formulation substantially impedes the progress of clinical development of PRI-724 [31].

## 5. Conclusions

To summarize, the study shows that targeting the CBP/β-catenin interaction with PRI-724 effectively downregulates survivin and promotes apoptosis in melanoma cells, especially in those resistant to trametinib, a MEK inhibitor. Our findings based on transcriptomic, immunoblotting, and bioinformatic analyses revealed that a high survivin level might be an indicator of poor prognosis for melanoma patients. The survivin level in drug-naïve and resistant melanoma cells might also determine the efficacy of PRI-724 to induce apoptosis and invasiveness. However, the potential use of survivin as a biomarker requires further investigation.

## Data Availability

The original data and findings presented in this study are available within the article and its Supplementary Materials. Raw and processed RNA-seq data were submitted to Gene Expression Omnibus and are available under accession number GSE301849. Please contact the corresponding author for any additional information or inquiries.

## Supplementary Materials

The following supporting information can be downloaded at: https://www.mdpi.com/article/10.3390/cells14211710/s1, Figure S1: Microphotographs of DMBC21, 21_TRAR, and 21_PLXR cells exposed to PRI-724 at 0.3 and 0.63 μM for 24–72 h; Figure S2: Percentages of propidium iodide (PI)-positive melanoma cells in untreated DMBC21, 21_TRAR, and 21_PLXR cell populations and after 48 h of treatment with PRI-724 at 0.63 µM. Single staining with PI was applied. Results of representative experiments are shown; Figure S3: RNA-seq analysis of transcriptomic data from DMBC21, 21_TRAR, and 21_PLXR cells, submitted to GEO as the GSE301849 dataset. The FPKMs of *BIRC5*, *CCND1*, *MITF*, *CDKN1A*, and *MYC* are shown as z-scores, representing the relative expression of each gene across all three cell lines; Table S1: Annotated list of missense mutations identified in DMBC21, 21_PLXR, and 21_TRAR melanoma cell lines using the Polyphen-2 (Polymorphism Phenotyping v2) tool [49] and predicted functional impact using the SIFT (Sorting Intolerant From Tolerant) tool. SIFT scores range from 0 to 1, with values <0.05 indicating substitutions predicted to be deleterious. PolyPhen-2 scores also range from 0 to 1 and mutations are classified as benign (values 0.000–0.449), possibly damaging (values 0.450–0.959), and probably damaging (values 0.960–1.000). The predictions are based on sequence conservation, structural context, and known protein features. Mutations are marked as homozygous (+/+) or heterozygous (+/−); Table S2: Top 150 genes upregulated in HNSCC samples after treatment with ICG-001. Data were reanalyzed from the GSE95704 dataset. Highlighted in red are genes linked to melanoma drug resistance; Table S3: Top 150 genes downregulated in HNSCC samples after treatment with ICG-001. Data were reanalyzed from the GSE95704 dataset. Highlighted in red are genes linked to melanoma drug resistance.

## Abbreviations

The following abbreviations are used in this manuscript:

AJCC	American Joint Committee on Cancer
bFGF	Basic fibroblast growth factor
BIRC5	Baculoviral inhibitor of apoptosis repeat-containing 5
BP	Biological process
BRAF	B-Raf murine sarcoma viral oncogene homolog B
CBP	CREB-binding protein
CC	Cellular component
CI	Confidence interval
DMBC	Department of Molecular Biology of Cancer
DMEM	Dulbecco’s modified Eagle’s medium
EGF	Epidermal growth factor
EGFR	Epidermal growth factor receptor
GCTs	Testicular germ cell tumors
GEO	Gene Expression Omnibus
GEPIA2	Gene Expression Profiling Interactive Analysis 2
GO	Gene ontology
GTEx	Genotype-Tissue Expression
HBSS	Hanks’ balanced salt solution
HR	Hazard ratio
HRP	Horseradish peroxidase
HSP-90	Heat shock protein-90
IAP	Inhibitor of apoptosis
ICIs	Immune checkpoint inhibitors
KEGG	Kyoto Encyclopedia of Genes and Genomes
MF	Molecular function
MI	Mitotic index
MMC	Mitomycin C
NES	Normalized enrichment score
ORA	Over-representation analysis
OSCC	Oral squamous cell carcinoma
PBS	Phosphate-buffered saline
PET	Polyethylene terephthalate
PI	Propidium iodide
PI3K/Akt	Phosphatidylinositol 3-kinase/protein kinase B
PreCOG	Prediction of Clinical Outcomes from Genomic Profiles
SCM	Stem cell medium
RWD	Relative wound density
SKCM	Skin cutaneous melanoma
STAT3	Signal transducer and activator of transcription 3
TCGA	The Cancer Genome Atlas
TKI	Tyrosine kinase inhibitor
YAP	Yes-associated protein
