# Supplementary material for: Cellular and Molecular Effects of Targeting the CBP/β-Catenin Interaction with PRI-724 in Melanoma Cells, Drug-Naïve and Resistant to Inhibitors of BRAFV600 and MEK1/2
Source: Cells. 2025 Oct 31;14(21):1710. doi: 10.3390/cells14211710 (PMC12609419; doi:10.3390/cells14211710)
Supplement: Supplementary file 1 [file cells-14-01710-s001.zip › cells-3908076-supplementary.pdf]

Supplementary Materials

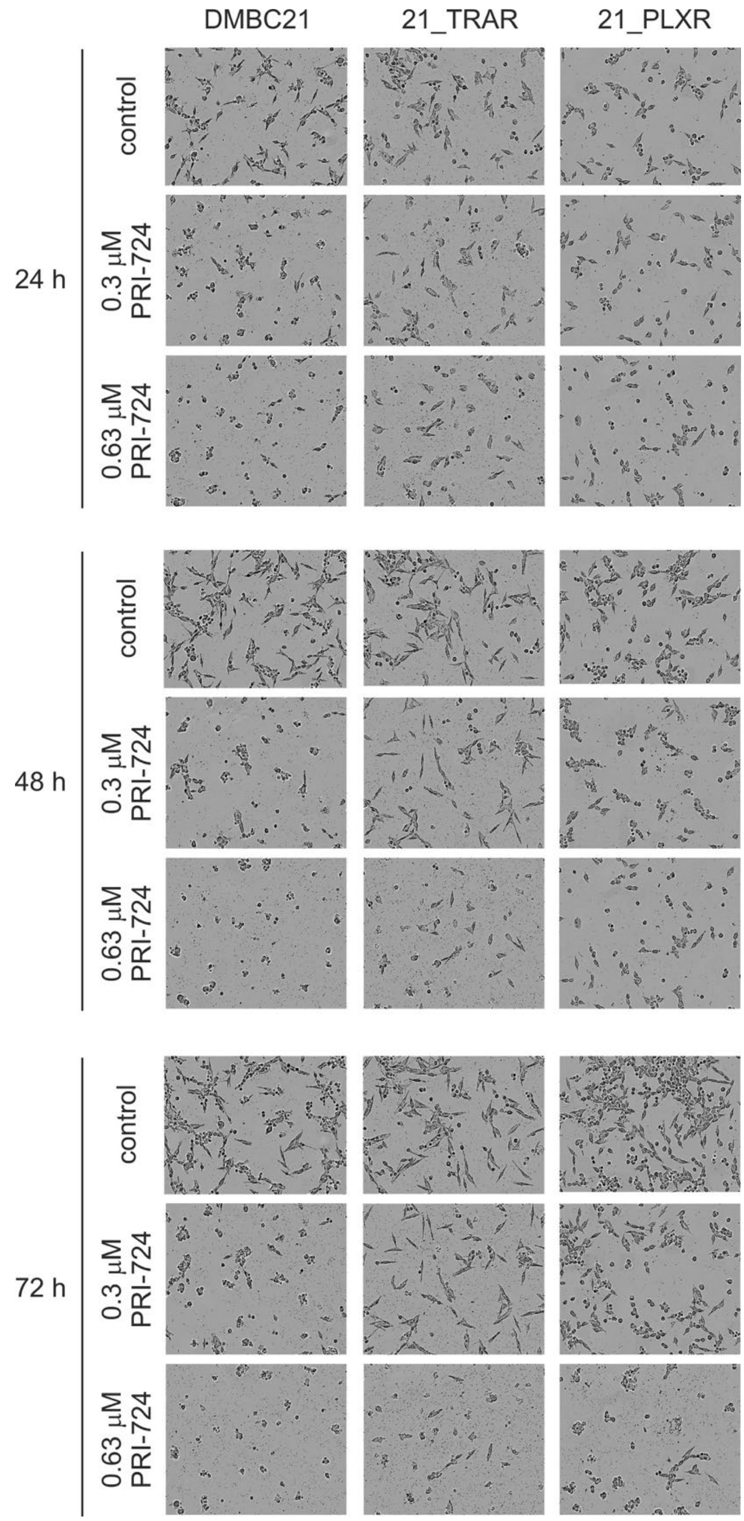

Figure S1. Microphotographs of DMBC21, 21_TRAR, and 21_PLXR cells exposed to PRI-724 at 0.3 and 0.63 µM for 24–72 h.

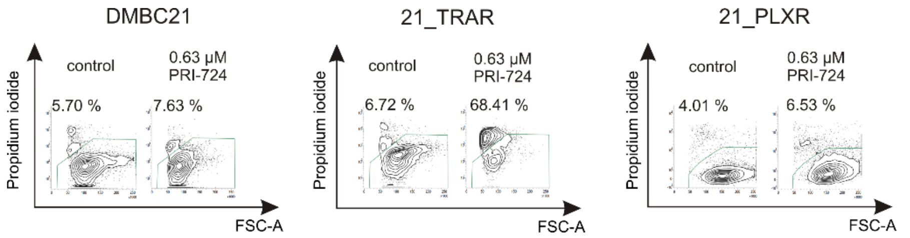

Figure S2. Percentages of propidium iodide (PI)-positive melanoma cells in untreated DMBC21, 21_TRAR, and 21_PLXR cell populations and after 48 h of treatment with PRI-724 at 0.63 µM. Single staining with PI was applied. Results of representative experiments are shown.

Table S1. Annotated list of missense mutations identified in DMBC21, 21_PLXR, and 21_TRAR melanoma cell lines using the Polyphen-2 (Polymorphism Phenotyping v2) tool [49] and predicted functional impact using the SIFT (Sorting Intolerant From Tolerant) tool. SIFT scores range from 0 to 1, with values <0.05 indicating substitutions predicted to be deleterious. PolyPhen-2 scores also range from 0 to 1 and mutations are classified as benign (values 0.000-0.449), possibly damaging (values 0.450-0.959), and probably damaging (values 0.960-1.000). The predictions are based on sequence conservation, structural context, and known protein features. Mutations are marked as homozygous (+/+) or heterozygous (+/-).

gene symbol	cell line						additional notes
	DMBC21		21_TRAR		21_PLXR		
	Polyphen-2 score	SIFT score	Polyphen-2 score	SIFT score	Polyphen-2 score	SIFT score	
ALK			I1461V (+/+)<br>0.000 (benign)	0.13 (tolerated)	I1461V (+/+)<br>0.000 (benign)	0.13 (tolerated)	MAPK pathway
ANKRD11	D2376E (+/+)<br>0.010 (benign)	0.00† (deleterious)	D2376E (+/+)<br>0.010 (benign)	0.00† (deleterious)	D2376E (+/+)<br>0.010 (benign)	0.00† (deleterious)	chromatin regulator
APC	V1822D (+/-)<br>0.000 (benign)	0.48 (tolerated)	V1822D (+/-)<br>0.000 (benign)	0.48 (tolerated)	V1822D (+/-)<br>0.000 (benign)	0.48 (tolerated)	WNT/β -catenin pathway
ARID1B					Y1427* (+/-)<br>stop gained		WNT/β -catenin pathway
ARID4A					N724S (+/+)<br>0.003 (benign)	0.00† (deleterious)	pRb pathway
					T779A (+/+)		

			0.000 (benign) 0.00† (deleterious)	
ASXL1	L815P +/+<br>0.000 (benign) 0.26 (tolerated)	L815P +/+<br>0.000 (benign) 0.26 (tolerated)	L815P +/+<br>0.000 (benign) 0.26 (tolerated)<br>G704R (+/-)<br>0.986 (probably damaging) 0.00† (deleterious)	Polycomb group protein
ATM	N1938S (+/+)<br>0.000 (benign) 0.00† (deleterious)	N1938S (+/+)<br>0.000 (benign) 0.00† (deleterious)	N1938S (+/+)<br>0.000 (benign) 0.00† (deleterious)	cell cycle and senescence
ATR	R2425Q (+/-)<br>0.000 (benign) 0.26 (tolerated)<br>M211T (+/-)<br>0.000 (benign) 0.00† (deleterious)	R2425Q (+/-)<br>0.000 (benign) 0.26 (tolerated)<br>M211T (+/-)<br>0.000 (benign) 0.00† (deleterious)	R2425Q (+/-)<br>0.000 (benign) 0.26 (tolerated)<br>M211T (+/+)<br>0.000 (benign) 0.00† (deleterious)	cell cycle and senescence
ATXN2	Q188 (+/+)<br>disruptive inframe deletion<br>L107V (+/-)<br>0.000 (benign) 0.95 (tolerated)	Q188 (+/+)<br>disruptive inframe deletion<br>L107V (+/-)<br>0.000 (benign) 0.95 (tolerated)	Q188 (+/+)<br>disruptive inframe deletion<br>L107V (+/+)<br>0.000 (benign) 0.95 (tolerated)	PI3K pathway
AXL	N266D (+/+)<br>0.000 (benign) 0.81 (tolerated)	N266D (+/+)<br>0.000 (benign) 0.81 (tolerated)	N266D (+/+)<br>0.000 (benign) 0.81 (tolerated)	receptor tyrosine kinase
BCL11B			V690M (+/-)<br>0.082 (benign) 0.01† (deleterious)	NF-κB signaling
BCORL1	F111L (+/+)<br>0.000 (benign) 1.00 (tolerated)	F111L (+/+)<br>0.000 (benign) 1.00 (tolerated)	F111L (+/+)<br>0.000 (benign) 1.00 (tolerated)	transcriptional corepressor
BRAF	V600E (+/-)	V600E (+/-)	V600E (+/+)	MAPK pathway

	0.971 (probably damaging)	0.00 (deleterious)	0.971 (probably damaging)	0.00 (deleterious)	0.971 (probably damaging)	0.00 (deleterious)	
BRCA1					S1634G (+/-)		DNA repair, tumor suppressor
					0.002 (benign)	0.03† (deleterious)	
					K1183R (+/-)		
					0.000 (benign)	0.98 (tolerated)	
					E1038G (+/-)		
					0.012 (benign)	0.00† (deleterious)	
					P871L (+/-)		
					0.000 (benign)	1.00 (tolerated)	
BRCA2	V2466A (+/+)		V2466A (+/+)		V2466A (+/+)		DNA repair, tumor suppressor
	0.000 (benign)	0.75 (tolerated)	0.000 (benign)	0.75 (tolerated)	0.000 (benign)	0.75 (tolerated)	
	T1915M (+/-)		T1915M (+/+)		T1915M (+/-)		
	0.000 (benign)	0.04† (deleterious)	0.000 (benign)	0.04* (deleterious)	0.000 (benign)	0.04† (deleterious)	
BRIP1	S919P (+/-)		S919P (+/+)		S919P (+/+)		DNA-dependent ATPase, 5' to 3' DNA helicase
	0.000 (benign)	0.21 (tolerated)	0.000 (benign)	0.21 (tolerated)	0.000 (benign)	0.21 (tolerated)	
CCNA2	I163V (+/-)		I163V (+/-)		I163V (+/+)		cell cycle
	0.000 (benign)	0.88 (tolerated)	0.000 (benign)	0.88 (tolerated)	0.000 (benign)	0.88 (tolerated)	
CCND3					S259A (+/+)		cell cycle
					0.000 (benign)	0.09 (tolerated)	
CCNH	V270A (+/-)		V270A (+/-)		V270A (+/-)		cell cycle
	0.965 (probably damaging)	0.16 (tolerated)	0.965 (probably damaging)	0.16 (tolerated)	0.965 (probably damaging)	0.16 (tolerated)	
CDKN1B					V109G (+/-)		cell cycle
					0.047 (benign)	0.00† (deleterious)	

CDKN2A					E69* (+/+) stop gained<br>E61* (+/+) stop gained		cell cycle
CHEK1	I471V (+/+)		I471V (+/+)		I471V (+/+)		DNA repair
	0.000 (benign)	1.00 (tolerated)	0.000 (benign)	1.00 (tolerated)	0.000 (benign)	1.00 (tolerated)	
CIITA	Q901R (+/+)		Q901R (+/+)		Q901R (+/+)		master regulator of MHC class II gene transcription
	0.745 (possibly damaging)	0.00† (deleterious)	0.745 (possibly damaging)	0.00† (deleterious)	0.745 (possibly damaging)	0.00† (deleterious)	
CTLA4	T17A (+/-)		T17A (+/-)		T17A (+/-)		immune checkpoint
	0.007 (benign)	0.05 (tolerated)	0.007 (benign)	0.05 (tolerated)	0.007 (benign)	0.05 (tolerated)	
CTR9					S477* (+/-) stop gained		RNA polymerase II complex binding
DDX53	M381I (+/-)		M381I (+/-)		M381I (+/+)		ATP-dependent RNA helicase
	0.999 (probably damaging)	0.00 (deleterious)	0.999 (probably damaging)	0.00 (deleterious)	0.999 (probably damaging)	0.00 (deleterious)	
DUSP6	V114L (+/+)		V114L (+/+)		V114L (+/+)		MAPK pathway
	0.780 (possibly damaging)	0.22 (tolerated)	0.780 (possibly damaging)	0.22 (tolerated)	0.780 (possibly damaging)	0.22 (tolerated)	
DUSP16					V366M (+/-)		MAPK pathway
					0.005 (benign)	0.02† (deleterious)	
E2F3					D148N (+/-)		oncogenic transcription factor
					0.915 (possibly damaging)	0.11 (tolerated)	
ECT2L					K676fs +(/-)<br>frameshift variant		Rho protein signal transduction
EGFR	R521K (+/-)		R521K (+/-)		R521K (+/-)		transmembrane signaling receptor
	0.000 (benign)	0.84 (tolerated)	0.000 (benign)	0.84 (tolerated)	0.000 (benign)	0.84 (tolerated)	
EP300					I997V (+/+)		histone acetyltransferase
					0.000 (benign)	1.00 (tolerated)	

			Q2223P (+/-)<br>0.000 (benign) 0.02† (deleterious)	and transcriptional activator
EP400	Q2742 (+/-) disruptive inframe insertion	Q2742 (+/-) disruptive inframe insertion	Q2742 (+/-) disruptive inframe insertion	DNA helicase, transcriptional activator
EPHA3	W924R (+/-)<br>0.000 (benign) 0.30 (tolerated)	W924R (+/-)<br>0.000 (benign) 0.30 (tolerated)	W924R (+/-)<br>0.000 (benign) 0.30 (tolerated)	receptor tyrosine kinase
ERBB2	P1170A (+/+)<br>0.953 (possibly damaging) 0.06 (tolerated)	P1170A (+/+)<br>0.953 (possibly damaging) 0.06 (tolerated)	P1170A (+/+)<br>0.953 (possibly damaging) 0.06 (tolerated)	transmembrane receptor protein tyrosine kinase
ERCC2	D312N (+/-)<br>0.016 (benign) 0.51 (tolerated)<br>K751Q (+/-)<br>0.000 (benign) 0.00† (deleterious)	D312N (+/-)<br>0.016 (benign) 0.51 (tolerated)<br>K751Q (+/-)<br>0.000 (benign) 0.00† (deleterious)	D312N (+/-)<br>0.016 (benign) 0.51 (tolerated)<br>K751Q (+/-)<br>0.000 (benign) 0.00† (deleterious)	DNA repair
FANCA			T266A (+/+)<br>0.000 (benign) 1.00 (tolerated)	DNA repair
FANCD2	N405S (+/-)<br>0.014 (benign) 1.00 (tolerated)	N405S (+/-)<br>0.014 (benign) 1.00 (tolerated)	N405S (+/-)<br>0.014 (benign 1.00 (tolerated)	DNA repair
FAT1	K4059N (+/+)<br>0.000 (benign) 0.85 (tolerated)<br>Q2933P (+/+)<br>0.000 (benign) 1.00 (tolerated)<br>R1064G (+/+)<br>0.000 (benign) 1.00 (tolerated)<br>V862L (+/+)<br>0.000 (benign) 1.00 (tolerated)	K4059N (+/+)<br>0.000 (benign) 0.85 (tolerated<br>Q2933P (+/+)<br>0.000 (benign) 1.00 (tolerated)<br>R1064G (+/+)<br>0.000 (benign) 1.00 (tolerated)<br>V862L (+/+)<br>0.000 (benign) 1.00 (tolerated)	K4059N (+/+)<br>0.000 (benign) 0.85 (tolerated)<br>Q2933P (+/+)<br>0.000 (benign) 1.00 (tolerated)<br>R1064G (+/+)<br>0.000 (benign) 1.00 (tolerated)<br>V862L (+/+)<br>0.000 (benign) 1.00 (tolerated)	cellular polarization

	F614L (+/+)		F614L (+/+)		F614L (+/+)		
	0.000 (benign)	0.27 (tolerated)	0.000 (benign)	0.27 (tolerated)	0.000 (benign)	0.27 (tolerated)	
	S404R (+/+)		S404R (+/+)		S404R (+/+)		
	0.032 (benign)	0.48 (tolerated)	0.032 (benign)	0.48 (tolerated)	0.032 (benign)	0.48 (tolerated)	
	V482I (+/+)		V482I (+/+)		V482I (+/+)		
	0.009 (benign)	0.00[†] (deleterious)	0.009 (benign)	0.00[†] (deleterious)	0.009 (benign)	0.00[†] (deleterious)	
FBXW7					V418M (+/-)		ubiquitin protein ligase
					0.972 (probably damaging)	0.15 (tolerated)	
FGFR4					G388R (+/-)		cell surface receptor protein tyrosine kinase
					0.998 (probably damaging)	0.10 (tolerated)	
FOXM1			P681S (+/-)				transcription factor
			0.000 (benign)	0.01[†] (deleterious)			
HNF1A	I27L (+/-)		I27L (+/-)		I27L (+/-)		transcription factor
	0.025 (benign)	0.34 (tolerated)	0.025 (benign)	0.34 (tolerated)	0.025 (benign)	0.34 (tolerated)	
	S487N (+/-)		S487N (+/-)		S487N (+/-)		
	0.014 (benign)	0.47 (tolerated)	0.014 (benign)	0.47 (tolerated)	0.014 (benign)	0.47 (tolerated)	
IGFBP7			L11F (+/+)		L11F (+/+)		senescence and autophagy
			0.005 (benign)	0.00[†] (deleterious)	0.005 (benign)	0.00[†] (deleterious)	
INPPL1					L632I (+/-)		PI3K pathway
					0.996 (probably damaging)	0.04[†] (deleterious)	
KDR	Q472H (+/-)		Q472H (+/-)		Q472H (+/-)		VEGF signaling
	0.003 (benign)	0.18 (tolerated)	0.003 (benign)	0.18 (tolerated)	0.003 (benign)	0.18 (tolerated)	
	V297I (+/-)		V297I (+/-)		V297I (+/-)		

	1.000 (probably damaging)	0.03 (deleterious)	1.000 (probably damaging)	0.03 (deleterious)	1.000 (probably damaging)	0.03 (deleterious)	
KMT2A	A30G (+/+)<br>0.953 (possibly damaging)	0.00[†] (deleterious)	A30G (+/+)<br>0.953 (possibly damaging)	0.00[†] (deleterious)	A30G (+/+)<br>0.953 (possibly damaging)	0.00[†] (deleterious)	lysine methyltransferase, transcriptional coactivator
KMT2B	R1021fs (+/+)<br>frameshift variant		R1021fs (+/+)<br>frameshift variant		R1021fs (+/+)<br>frameshift variant		lysine methyltransferase, transcriptional coactivator
KMT2C					C988F (+/-)<br>0.999 (probably damaging)	0.00[†] (deleterious)	lysine methyltransferase, transcriptional coactivator
					T316S (+/-)<br>0.684 (possibly damaging)	0.16 (tolerated)	
					L291F (+/-)<br>1.000 (probably damaging)	0.00[†] (deleterious)	
					Y816X +/-<br>frameshift variant		
MC1R					R151C (+/+)<br>1.000 (probably damaging)	0.00 (deleterious)	melanocyte development and pigmentation
	V60L (+/-)<br>0.988 (probably damaging)	0.27 (tolerated)	V60L (+/-)<br>0.988 (probably damaging)	0.27 (tolerated)	V60L (+/-)<br>0.988 (probably damaging)	0.27 (tolerated)	
MED12					H2116 (+/+)<br>disruptive inframe insertion		RNA polymerase II – dependent gene expression
MEN1	T546A (+/+)<br>0.000 (benign)	0.85 (tolerated)	T546A (+/+)<br>0.000 (benign)	0.85 (tolerated)	T546A (+/+)<br>0.000 (benign)	0.85 (tolerated)	lysine methyltransferase,

							transcriptional coactivator
MGA	P1523A (+/+)		P1523A (+/+)		P1523A (+/+)		transcription factor
	0.189 (benign)	0.58 (tolerated)	0.189 (benign)	0.58 (tolerated)	0.189 (benign)	0.58 (tolerated)	
MKI67	I2101T (+/-)		I2101T (+/-)		I2101T (+/+)		cell proliferation
	0.013 (benign)	0.06 (tolerated)	0.013 (benign)	0.06 (tolerated)	0.013 (benign)	0.06 (tolerated)	
	N104S (+/+)		N104S (+/+)		N104S (+/+)		
	0.242 (benign)	0.06 (tolerated)	0.242 (benign)	0.06 (tolerated)	0.242 (benign)	0.06 (tolerated)	
	T2868S (+/-)		T2868S (+/-)		T2868S (+/-)		
	0.820 (possibly damaging)	0.59 (tolerated)	0.820 (possibly damaging)	0.59 (tolerated)	0.820 (possibly damaging)	0.59 (tolerated)	
	R2786Q (+/-)		R2786Q (+/-)		R2786Q (+/-)		
	0.743 (possibly damaging)	0.36 (tolerated)	0.743 (possibly damaging)	0.36 (tolerated)	0.743 (possibly damaging)	0.36 (tolerated)	
	E1403V (+/-)		E1403V (+/-)		E1403V (+/-)		
	0.999 (probably damaging)	0.00[†] (deleterious)	0.999 (probably damaging)	0.00[†] (deleterious)	0.999 (probably damaging)	0.00[†] (deleterious)	
	K3217E (+/-)		K3217E (+/-)		K3217E (+/-)		
	0.000 (benign)	1.00 (tolerated)	0.000 (benign)	1.00 (tolerated)	0.000 (benign)	1.00 (tolerated)	
	T3150S (+/-)		T3150S (+/-)		T3150S (+/-)		
	0.000 (benign	1.00 (tolerated)	0.000 (benign	1.00 (tolerated)	0.000 (benign)	1.00 (tolerated)	
	N2363S (+/-)		N2363S (+/-)		N2363S (+/-)		
	0.000 (benign)	0.52 (tolerated)	0.000 (benign)	0.52 (tolerated)	0.000 (benign)	0.52 (tolerated)	
	G1042S (+/-)		G1042S (+/-)		G1042S (+/-)		
	0.955 (possibly damaging)	0.84 (tolerated)	0.955 (possibly damaging)	0.84 (tolerated)	0.955 (possibly damaging)	0.84 (tolerated)	
MLH1	I219V (+/-)		I219V (+/-)		I219V (+/-)		DNA repair

	0.015 (benign)	0.57 (tolerated)	0.015 (benign)	0.57 (tolerated)	0.015 (benign)	0.57 (tolerated)	
MSH3	A61-P63dup +/-		A61-P63dup +/-		A61-P63dup +/-		DNA repair
	inframe insertion		inframe insertion		inframe insertion		
	Q949R (+/+)		Q949R (+/+)		Q949R (+/+)		
	0.000 (benign)	1.00 (tolerated)	0.000 (benign)	1.00 (tolerated)	0.000 (benign)	1.00 (tolerated)	
	A1045T (+/-)		A1045T (+/-)		A1045T (+/-)		
	0.075 (benign)	0.57 (tolerated)	0.075 (benign)	0.57 (tolerated)	0.075 (benign)	0.57 (tolerated)	
MSH6	G39E (+/-)		G39E (+/-)		G39E (+/-)		DNA repair
	0.000 (benign)	0.11 (tolerated)	0.000 (benign)	0.11 (tolerated)	0.000 (benign)	0.11 (tolerated)	
MTOR					R2152C (+/-)		cell division and survival
					0.958 (probably damaging)	0.00 (deleterious)	
MYT1	T782S (+/-)		T782S (+/-)		T782S (+/-)		cell differentiation
	0.001 (benign)	0.36 (tolerated)	0.001 (benign)	0.36 (tolerated)	0.001 (benign)	0.36 (tolerated)	
NBN					E185Q (+/-)		DNA repair
					0.000 (benign)	0.65 (tolerated)	
NOTCH2					F1209V +/-		Notch signaling
					0.939 (possibly damaging)	0.01 (deleterious)	
					N46S (+/-)		
					0.713 (possibly damaging)	0.14 (tolerated)	
					E38K (+/-)		
					0.001 (benign)	0.50 (tolerated)	
			C19W (+/-)		C19W (+/-)		
			0.001 (benign)	0.02† (deleterious)	0.001 (benign)	0.02† (deleterious)	

		A21T (+/-)<br>0.028 (benign) 0.42 (tolerated)<br>P6fs +/-<br>frameshift variant		
NOTCH3	A2223V (+/+)<br>0.001 (benign) 0.13 (tolerated)	A2223V (+/+)<br>0.001 (benign) 0.13 (tolerated)	A2223V (+/+)<br>0.001 (benign) 0.13 (tolerated)<br>C1826F (+/-)<br>0.829 (possibly damaging) 0.69 (tolerated)	Notch signaling
PIK3R2	S313P (+/+)<br>0.000 (benign) 1.00 (tolerated)<br>S234R (+/+)<br>0.000 (benign) 0.50 (tolerated)	S313P (+/+)<br>0.000 (benign) 1.00 (tolerated)<br>S234R (+/+)<br>0.000 (benign) 0.50 (tolerated)	S313P (+/+)<br>0.000 (benign) 1.00 (tolerated)<br>S234R (+/+)<br>0.000 (benign) 0.50 (tolerated)	PI3K pathway
PIK3R3		N283K (+/+)<br>0.000 (benign) 1.00 (tolerated)	N283K (+/+)<br>0.000 (benign) 1.00 (tolerated)<br>M295X (+/-)<br>frameshift variant	PI3K pathway
PMS2	K541E (+/-)<br>0.000 (benign) 0.37 (tolerated)	K541E (+/-)<br>0.000 (benign) 0.37 (tolerated)	K541E (+/-)<br>0.000 (benign) 0.37 (tolerated)<br>P470S (+/-)<br>0.002 (benign) 1.00 (tolerated)	DNA repair
PPP2R1A			E332D<br>0.000 (benign) 1.00 (tolerated)	protein serine/threonine phosphatase activity
PRDM1			G74S (+/-)<br>0.000 (benign) 0.00† (deleterious)	cell differentiation and immune suppression
PTCH1	P1315L (+/-)	P1315L (+/-)	P1315L (+/-)	SHH pathway

	0.906 (possibly damaging)	0.00† (deleterious)	0.906 (possibly damaging)	0.00† (deleterious)	0.906 (possibly damaging)	0.00† (deleterious)	
PTPRD	T781A (+/-)		T781A (+/-)		T781A (+/-)		STAT3 signaling
	0.001 (benign)	0.37 (tolerated)	0.001 (benign)	0.37 (tolerated)	0.001 (benign)	0.37 (tolerated)	
					G272R (+/-)		
					0.976 (probably damaging)	0.08 (tolerated)	
PTPRS	C1457R (+/+)		C1457R (+/+)		C1457R (+/+)		STAT3 signaling
	0.000 (benign)	0.44 (tolerated)	0.000 (benign)	0.44 (tolerated)	0.000 (benign)	0.44 (tolerated)	
PTPRT	A29P (+/+)		A29P (+/+)		A29P (+/+)		STAT3 signaling
	0.008 (benign)	0.04† (deleterious)	0.008 (benign)	0.04† (deleterious)	0.008 (benign)	0.04† (deleterious)	
RASGRF2					S753P (+/-)		ERK pathway
					0.002 (benign)	0.00† (deleterious)	
RASGRP3					T393A (+/-)		ERK pathway
					0.131 (benign)	0.62 (tolerated)	
RET					G691S (+/+)		neural development
					0.062 (benign)	0.01† (deleterious)	
RICTOR	S837F (+/+)		S837F (+/+)		S837F (+/+)		cell division and survival
	0.002 (benign)	0.19 (tolerated)	0.002 (benign)	0.19 (tolerated)	0.002 (benign)	0.19 (tolerated)	
RNF43					P231L (+/+)		WNT/β-catenin pathway
					0.760 (probably damaging)	0.04† (deleterious)	
	I47V (+/-)		I47V (+/-)		I47V (+/-)		
	0.000 (benign)	1.000 (tolerated)	0.000 (benign)	1.000 (tolerated)	0.000 (benign)	1.000 (tolerated)	
	L418M (+/-)		L418M (+/-)		L418M (+/-)		

	0.948 (possibly damaging)	0.00† (deleterious)	0.948 (possibly damaging)	0.00† (deleterious)	0.948 (possibly damaging)	0.00† (deleterious)	
	R343H (+/-)		R343H (+/-)		R343H (+/-)		
	0.999 (probably damaging)	0.02† (deleterious)	0.999 (probably damaging)	0.02† (deleterious)	0.999 (probably damaging)	0.02† (deleterious)	
ROS1	D2213N (+/+)		D2213N (+/+)		D2213N (+/+)		cell division and differentiation
	0.003 (benign)	0.98 (tolerated)	0.003 (benign)	0.98 (tolerated)	0.003 (benign)	0.98 (tolerated)	
	S2229C (+/+)		S2229C (+/+)		S2229C (+/+)		
	0.000 (benign)	1.00 (tolerated)	0.000 (benign)	1.00 (tolerated)	0.000 (benign)	1.00 (tolerated)	
	K2228Q (+/+)		K2228Q (+/+)		K2228Q (+/+)		
	0.000 (benign)	0.30 (tolerated)	0.000 (benign)	0.30 (tolerated)	0.000 (benign)	0.30 (tolerated)	
RPS6KA4	S758A +/-		S758A +/-		S758A +/-		MAPK pathway
	0.000 (benign)	0.00† (deleterious)	0.000 (benign)	0.00† (deleterious)	0.000 (benign)	0.00† (deleterious)	
RTEL1	Q1042H (+/-)		Q1042H (+/-)		Q1042H (+/-)		DNA replication and repair
	0.000 (benign)	0.24 (tolerated)	0.000 (benign)	0.24 (tolerated)	0.000 (benign)	0.24 (tolerated)	
SETD2	P1962L (+/-)		P1962L (+/-)		P1962L (+/-)		histone methyltransferase
	0.000 (benign)	0.00† (deleterious)	0.000 (benign)	0.00† (deleterious)	0.000 (benign)	0.00† (deleterious)	
SHQ1	S489N (+/-)		S489N (+/-)		S489N (+/+)		RNA processing
	0.000 (benign)	0.46 (tolerated)	0.000 (benign)	0.46 (tolerated)	0.000 (benign)	0.46 (tolerated)	
					F72C (+/-)		
					1.000 (probably damaging)	0.02† (deleterious)	
SOS1					R647M (+/-)		MAPK pathway
					0.999 (probably damaging)	0.00† (deleterious)	

SPEN					L2650 (+/-) disruptive inframe deletion		chromatin remodeling
TET1	D162G (+/-)		D162G (+/-)		D162G (+/-)		DNA demethylation
	0.295 (benign)	0.42 (tolerated)	0.295 (benign)	0.42 (tolerated)	0.295 (benign)	0.42 (tolerated)	
	I1123M (+/+)		I1123M (+/+)		I1123M (+/+)		
	0.070 (benign)	0.01[†] (deleterious)	0.070 (benign)	0.01[†] (deleterious)	0.070 (benign)	0.01[†] (deleterious)	
					V128F (+/+)		
					0.845 (possibly damaging)	0.08 (tolerated)	
	I1762V (+/-)		I1762V (+/-)		I1762V (+/+)		
	0.012 (benign)	0.89 (tolerated)	0.012 (benign)	0.89 (tolerated)	0.012 (benign)	0.89 (tolerated)	
TOP1					H81Y (+/-)		DNA topoisomerase
					0.273 (benign)	0.00[†] (deleterious)	
TP53	P72R (+/-)		P72R (+/-)		P72R (+/-)		cell cycle and senescence
	0.083 (benign)	0.02[†] (deleterious)	0.083 (benign)	0.02[†] (deleterious)	0.083 (benign)	0.02[†] (deleterious)	
TP53BP1					K1141Q (+/-)		DNA repair
					0.181 (benign)	0.16 (tolerated)	
TSC1	M322T (+/-)		M322T (+/-)		M322T (+/-)		cell division and survival
	0.000 (benign)	0.66 (tolerated)	0.000 (benign)	0.66 (tolerated)	0.000 (benign)	0.66 (tolerated)	
					I346N (+/-)		
					0.408 (benign)	0.21 (tolerated)	
WNK1	T1316P (+/+)		T1316P (+/+)		T1316P (+/+)		transmembrane ion transport
	0.800 (possibly damaging)	0.00* (deleterious)	0.800 (possibly damaging)	0.00* (deleterious)	0.800 (possibly damaging)	0.00[†] (deleterious)	
					F741fs +/+ frameshift variant		

ZFHX3	V777A (+/+)	0.002 (benign)	0.02† (deleterious)	V777A (+/+)	0.002 (benign)	0.02† (deleterious)	circadian clock
				S72A (+/+)	0.006 (benign)	0.09 (tolerated)	

†Low-confidence SIFT prediction; this substitution may have been predicted to affect protein function due to limited sequence diversity in the alignment, therefore, the prediction result should be interpreted with caution.

Table S2

Top 150 genes upregulated in HNSCC samples after treatment with ICG-001. Data were reanalyzed from the GSE95704 dataset. Highlighted in red are genes linked to melanoma drug resistance.

Gene	log_2 (Fold Change)	p-value	FDR
CYP1A1	4.481	1.85E-06	6.20E-04
GDF15	2.332	8.23E-04	1.92E-02
TRIB3	2.029	9.69E-08	1.04E-04
TMEM156	1.989	1.81E-03	3.05E-02
MSC	1.934	1.22E-03	2.43E-02
CYP1B1	1.814	1.90E-04	7.95E-03
ALOX5AP	1.755	7.85E-07	3.52E-04
CHAC1	1.688	6.51E-06	1.40E-03
NCF2	1.586	1.27E-06	5.19E-04
SESN2	1.581	7.45E-11	1.00E-06
KLHL24	1.580	4.68E-04	1.37E-02
UNC5B	1.526	8.29E-04	1.92E-02
PNRC1	1.487	4.22E-04	1.28E-02
DDIT3	1.474	3.03E-04	1.05E-02
GULP1	1.473	1.95E-05	2.23E-03
PCK2	1.421	9.60E-10	6.45E-06
SCN9A	1.414	3.95E-03	4.96E-02
IL20RB	1.401	7.18E-05	4.67E-03
TCP11L2	1.385	6.80E-04	1.70E-02
ARHGEF2	1.374	2.42E-08	6.51E-05
SLC1A4	1.368	3.47E-04	1.14E-02
DMGDH	1.354	3.02E-05	2.88E-03

Table S3

Top 150 genes downregulated in HNSCC samples after treatment with ICG-001. Data were reanalyzed from the GSE95704 dataset. Highlighted in red are genes linked to melanoma drug resistance.

Gene	log_2 (Fold Change)	p-value	FDR
CPA4	-1.648	5.19E-06	1.21E-03
FST	-1.478	9.49E-06	1.59E-03
TYMS	-1.396	5.31E-06	1.21E-03
ARHGDIB	-1.322	4.45E-04	1.32E-02
SPC25	-1.264	4.84E-04	1.40E-02
CHRNB1	-1.263	4.38E-04	1.31E-02
CENPE	-1.245	9.52E-06	1.59E-03
GJA1	-1.240	1.45E-05	1.99E-03
GINS2	-1.204	6.34E-04	1.63E-02
UNG	-1.138	2.68E-03	3.91E-02
MYB	-1.124	6.71E-05	4.46E-03
HELLS	-1.122	8.04E-04	1.89E-02
OSR2	-1.111	5.65E-07	3.04E-04
CALB1	-1.094	5.07E-07	2.91E-04
CCNF	-1.088	1.18E-04	5.95E-03
DEPDC1B	-1.084	5.58E-04	1.50E-02
PLEKHA2	-1.068	9.18E-07	3.98E-04
SYT16	-1.064	1.18E-04	5.95E-03
ADAMTS1	-1.062	6.39E-05	4.34E-03
FAM83D	-1.061	2.46E-03	3.70E-02
CENPI	-1.027	9.61E-05	5.29E-03
BUB1	-1.026	1.30E-05	1.88E-03

ZNF765	1.322	7.45E-05	4.77E-03	LMNB1	-1.023	1.07E-03	2.23E-02
CCPG1	1.291	7.82E-04	1.86E-02	HJURP	-1.021	2.14E-03	3.38E-02
PORCN	1.288	3.84E-05	3.40E-03	MYBL2	-1.018	2.45E-03	3.70E-02
SLC7A11	1.272	1.63E-03	2.85E-02	CTNNAL1	-1.017	1.95E-03	3.18E-02
TPP1	1.271	9.43E-05	5.26E-03	MAMDC2	-1.013	6.21E-07	3.13E-04
TPRA1	1.243	2.42E-06	7.38E-04	SKP2	-1.012	1.21E-04	6.02E-03
LHFPL2	1.222	6.77E-05	4.48E-03	RGS2	-1.007	6.13E-05	4.29E-03
MKNK2	1.203	1.64E-06	5.80E-04	GTSE1	-0.992	1.31E-03	2.52E-02
TMC4	1.193	2.79E-05	2.76E-03	MKI67	-0.992	6.28E-04	1.62E-02
HBEGF	1.192	6.98E-08	1.04E-04	NEXN	-0.989	1.28E-03	2.49E-02
FUCA1	1.155	4.97E-04	1.41E-02	NCAPG	-0.986	2.06E-05	2.29E-03
STC2	1.149	5.48E-04	1.49E-02	CCNB2	-0.985	1.55E-03	2.78E-02
SQSTM1	1.141	1.35E-05	1.91E-03	MED27	-0.981	1.32E-03	2.54E-02
KYNU	1.126	3.83E-03	4.85E-02	AURKA	-0.980	3.46E-03	4.61E-02
C5AR1	1.117	1.54E-04	6.99E-03	SERPINE1	-0.975	2.56E-04	9.45E-03
ERN1	1.103	4.79E-07	2.91E-04	CLSPN	-0.973	8.66E-05	5.02E-03
PAPPA	1.098	1.72E-06	5.92E-04	NEIL3	-0.972	2.51E-03	3.74E-02
KLHDC7B	1.087	7.41E-04	1.79E-02	CNN3	-0.971	6.83E-06	1.40E-03
RBCK1	1.081	3.43E-08	7.69E-05	POLE2	-0.968	5.61E-08	9.74E-05
LTBP1	1.081	1.31E-05	1.88E-03	E2F8	-0.967	2.47E-04	9.31E-03
ABHD4	1.076	3.54E-06	9.52E-04	NUSAP1	-0.962	2.12E-04	8.58E-03
FUT3	1.067	1.47E-04	6.84E-03	KIF14	-0.960	8.18E-06	1.56E-03
ADCY7	1.056	3.43E-05	3.14E-03	RBMS3	-0.951	2.55E-03	3.81E-02
FADS3	1.054	3.08E-03	4.26E-02	DEPDC7	-0.946	3.74E-05	3.35E-03
SRXN1	1.048	1.07E-03	2.23E-02	CDKN2C	-0.946	2.84E-06	8.47E-04
DYSF	1.038	5.40E-05	4.01E-03	ASPM	-0.937	5.60E-05	4.07E-03
PLA2G4C	1.036	2.18E-03	3.44E-02	DNA2	-0.935	4.47E-06	1.13E-03
KRT7	1.031	2.74E-03	3.96E-02	GMNN	-0.933	2.53E-05	2.60E-03
CEBPG	1.020	2.67E-07	1.89E-04	ERCC6L	-0.930	6.27E-04	1.62E-02
NFE2L1	1.020	4.33E-05	3.55E-03	FUS	-0.928	5.78E-05	4.15E-03
PDE5A	1.017	2.22E-04	8.71E-03	NUF2	-0.922	1.29E-04	6.30E-03
TBL1X	1.015	7.02E-05	4.60E-03	ARHGAP29	-0.922	7.97E-05	4.81E-03
LMO4	1.014	1.05E-07	1.04E-04	MCM10	-0.919	2.35E-04	9.00E-03
ASNS	1.012	1.90E-04	7.95E-03	MAD2L1	-0.914	1.52E-04	6.91E-03
MGAT4B	1.010	6.29E-07	3.13E-04	ANXA9	-0.911	2.31E-03	3.60E-02
TSKU	1.010	2.31E-05	2.45E-03	CCNB1	-0.906	1.84E-03	3.07E-02
GCLC	1.005	6.97E-04	1.72E-02	UBE2T	-0.902	5.30E-06	1.21E-03

IL1RN	0.992	6.69E-07	3.21E-04	NDC80	-0.899	1.81E-04	7.74E-03
DDIT4	0.981	4.79E-04	1.39E-02	DLGAP5	-0.896	2.34E-04	9.00E-03
NDUFA4L2	0.969	5.45E-04	1.48E-02	CCNA2	-0.892	3.24E-04	1.09E-02
SGSM2	0.959	8.35E-06	1.56E-03	CDCA2	-0.891	1.92E-05	2.22E-03
ETV5	0.957	3.88E-03	4.89E-02	NRG1	-0.890	9.38E-04	2.07E-02
VEGFA	0.943	1.09E-04	5.73E-03	PLK1	-0.890	2.66E-03	3.89E-02
ORAI3	0.939	3.94E-04	1.23E-02	PSRC1	-0.888	1.60E-03	2.82E-02
HERPUD1	0.939	7.61E-05	4.80E-03	HSD17B11	-0.888	1.07E-03	2.23E-02
MORN4	0.936	1.83E-05	2.22E-03	OAS3	-0.887	1.12E-04	5.78E-03
APOL6	0.934	3.43E-03	4.59E-02	SPAG5	-0.884	3.49E-06	9.52E-04
TMEM53	0.925	5.40E-04	1.47E-02	ZWINT	-0.869	2.53E-05	2.60E-03
METRNL	0.900	9.83E-06	1.59E-03	NEK2	-0.865	3.26E-06	9.49E-04
C10orf55	0.898	3.97E-05	3.45E-03	ESPL1	-0.865	5.86E-05	4.17E-03
PHGDH	0.889	2.14E-04	8.63E-03	TOP2A	-0.859	9.46E-04	2.08E-02
ADAP1	0.883	1.24E-03	2.46E-02	GAS2L3	-0.859	1.47E-04	6.84E-03
PGPEP1	0.876	2.40E-03	3.66E-02	ANKRD22	-0.858	4.43E-04	1.32E-02
PGLS	0.872	1.39E-06	5.35E-04	TPX2	-0.854	2.58E-03	3.82E-02
PYGB	0.871	1.73E-05	2.18E-03	MCM3	-0.849	6.10E-04	1.59E-02
OSGIN1	0.861	2.73E-03	3.95E-02	TK1	-0.848	1.51E-04	6.90E-03
SLC17A5	0.859	1.50E-03	2.71E-02	NUAK2	-0.848	2.39E-03	3.65E-02
PLAU	0.857	7.16E-04	1.74E-02	SESTD1	-0.847	9.97E-05	5.40E-03
CTSO	0.856	2.85E-05	2.78E-03	EMP2	-0.839	1.18E-07	1.06E-04
TTLL1	0.849	4.65E-05	3.66E-03	HMMR	-0.839	2.18E-04	8.67E-03
UST	0.849	8.70E-04	1.97E-02	TMEM97	-0.837	4.03E-04	1.24E-02
TSPAN31	0.847	1.25E-05	1.86E-03	CDCA8	-0.836	6.33E-05	4.32E-03
GTPBP2	0.842	1.08E-04	5.73E-03	WDR76	-0.832	1.74E-04	7.57E-03
HBP1	0.842	2.58E-03	3.82E-02	RBM24	-0.832	3.95E-03	4.96E-02
PPARA	0.840	5.54E-05	4.07E-03	ARHGAP11A	-0.831	4.94E-04	1.41E-02
CD55	0.834	2.20E-08	6.51E-05	SHCBP1	-0.830	6.89E-06	1.40E-03
NFIL3	0.834	1.34E-03	2.56E-02	TACC3	-0.827	1.49E-05	1.99E-03
RAP1GAP	0.832	1.75E-04	7.57E-03	HERC5	-0.824	2.46E-07	1.84E-04
UPP1	0.827	1.08E-07	1.04E-04	KIF4A	-0.823	5.01E-06	1.20E-03
MYLK	0.827	6.21E-05	4.30E-03	SPAG1	-0.821	1.95E-03	3.18E-02
PGAP3	0.824	8.21E-06	1.56E-03	POLQ	-0.812	2.23E-04	8.74E-03
MOCOS	0.822	6.71E-05	4.46E-03	NCAPH	-0.807	4.34E-06	1.12E-03
VAT1	0.821	4.44E-05	3.60E-03	MNS1	-0.803	3.57E-03	4.68E-02
PLD3	0.821	2.60E-05	2.63E-03	RACGAP1	-0.796	2.95E-04	1.03E-02

GRN	0.820	4.10E-05	3.49E-03	TIPIN	-0.796	2.67E-03	3.90E-02
WIPI1	0.809	1.27E-03	2.48E-02	KIF11	-0.794	5.30E-05	3.97E-03
SH3PXD2B	0.805	5.71E-04	1.51E-02	NCAPG2	-0.794	3.02E-04	1.05E-02
KLHL5	0.805	5.57E-05	4.07E-03	CHAF1B	-0.794	3.69E-03	4.75E-02
OSCP1	0.802	9.65E-04	2.10E-02	CORO1C	-0.792	7.90E-05	4.80E-03
TIMP4	0.802	6.73E-06	1.40E-03	TRIP13	-0.791	1.31E-03	2.52E-02
C1RL	0.801	3.91E-04	1.22E-02	RAD54L	-0.789	3.63E-04	1.17E-02
CLYBL	0.790	4.88E-04	1.40E-02	RGS10	-0.789	1.10E-03	2.26E-02
STEAP3	0.783	4.41E-04	1.31E-02	FBXO5	-0.782	1.26E-03	2.48E-02
MMP15	0.783	1.39E-03	2.60E-02	CDCA3	-0.779	2.19E-03	3.46E-02
LONRF1	0.778	1.84E-03	3.07E-02	ATAD2	-0.776	1.39E-05	1.93E-03
HMGCL	0.778	9.82E-06	1.59E-03	GINS1	-0.773	1.07E-03	2.23E-02
PLSCR1	0.774	3.91E-03	4.92E-02	VRK1	-0.772	1.17E-05	1.81E-03
TBC1D20	0.770	1.23E-03	2.44E-02	KREMEN1	-0.766	1.90E-05	2.22E-03
FOSL2	0.768	2.47E-03	3.71E-02	CEP55	-0.765	5.20E-04	1.45E-02
LGALS3	0.764	4.39E-05	3.58E-03	RAD51AP1	-0.765	1.15E-04	5.88E-03
KRCC1	0.763	1.67E-05	2.13E-03	RFX7	-0.762	1.50E-05	1.99E-03
IFI30	0.758	3.86E-04	1.22E-02	TCOF1	-0.759	5.06E-04	1.43E-02
SLC3A2	0.756	7.48E-06	1.48E-03	ESCO2	-0.757	3.05E-03	4.25E-02
TUBE1	0.752	8.55E-04	1.95E-02	MELK	-0.749	1.05E-05	1.68E-03
RAB38	0.747	4.79E-06	1.19E-03	CKAP2L	-0.749	3.47E-03	4.61E-02
SEC11C	0.745	2.83E-03	4.03E-02	APAF1	-0.746	6.93E-05	4.57E-03
FA2H	0.740	3.44E-04	1.13E-02	GNB4	-0.741	2.41E-03	3.66E-02
ECH1	0.737	8.44E-06	1.56E-03	RRM1	-0.739	2.85E-05	2.78E-03
CBLB	0.735	9.01E-05	5.13E-03	DSCC1	-0.737	3.89E-04	1.22E-02
EPHX1	0.734	1.73E-03	2.98E-02	ANLN	-0.736	3.09E-03	4.28E-02
TSC22D1	0.733	3.88E-04	1.22E-02	HMGB2	-0.735	5.47E-05	4.04E-03
EIF4EBP1	0.733	1.35E-06	5.32E-04	KIFC1	-0.733	1.11E-03	2.27E-02
SLC35D2	0.732	8.48E-04	1.94E-02	LYAR	-0.731	5.26E-04	1.46E-02
SEL1L	0.731	7.13E-04	1.74E-02	RAD54B	-0.724	1.72E-04	7.56E-03
NR3C2	0.730	2.85E-03	4.05E-02	NCAPD3	-0.719	8.98E-06	1.59E-03
HOMEZ	0.729	2.06E-03	3.32E-02	DIAPH3	-0.717	2.59E-05	2.63E-03
DNAL4	0.726	2.16E-04	8.67E-03	EZH2	-0.708	7.14E-04	1.74E-02
ANXA4	0.725	2.25E-07	1.78E-04	RPL13A	-0.705	3.43E-03	4.59E-02
MAP1LC3B	0.721	3.32E-04	1.10E-02	PTPRZ1	-0.704	3.14E-04	1.07E-02
SPRY2	0.716	3.85E-04	1.22E-02	RRM2	-0.703	1.26E-05	1.86E-03
FZD7	0.715	2.64E-03	3.88E-02	BRIP1	-0.695	5.31E-04	1.46E-02

TPRG1L	0.705	1.78E-03	3.03E-02
RNPEPL1	0.701	4.09E-05	3.49E-03
CORO2A	0.700	4.12E-05	3.49E-03
SLC1A5	0.700	7.40E-07	3.43E-04
XPOT	0.699	9.64E-06	1.59E-03
NICN1	0.697	1.42E-03	2.62E-02
ITPR3	0.696	4.25E-05	3.54E-03
CTBS	0.694	4.14E-04	1.27E-02
CLIP4	0.687	1.20E-03	2.41E-02
HOXB9	0.685	9.35E-04	2.07E-02
MYO5B	0.683	1.10E-04	5.73E-03
GCC1	0.680	1.36E-04	6.51E-03
MAN2B2	0.680	1.42E-03	2.62E-02
NEU1	0.678	5.50E-04	1.49E-02
RAB9A	0.676	4.88E-04	1.40E-02
CYP2R1	0.676	3.52E-04	1.14E-02
CLCN7	0.675	7.83E-04	1.86E-02

FDR, false discovery rate

BIRC5	-0.692	1.31E-04	6.36E-03
S100A2	-0.692	3.76E-05	3.35E-03
PCNA	-0.692	4.50E-05	3.63E-03
SLC1A3	-0.691	1.40E-03	2.60E-02
TMPO	-0.691	3.55E-05	3.22E-03
INSIG1	-0.689	9.57E-06	1.59E-03
KIF20B	-0.687	9.64E-04	2.10E-02
GJC1	-0.687	2.71E-04	9.65E-03
KLHL13	-0.686	6.52E-04	1.65E-02
CD177	-0.685	8.31E-04	1.92E-02
ARID5B	-0.680	3.32E-06	9.49E-04
STMN1	-0.672	4.50E-04	1.33E-02
TRAF5	-0.668	4.54E-05	3.63E-03
STARD13	-0.667	3.97E-04	1.23E-02
ECT2	-0.664	1.80E-05	2.22E-03
PPP1CC	-0.664	1.37E-05	1.92E-03
HAS3	-0.664	2.71E-05	2.69E-03

FDR, false discovery rate

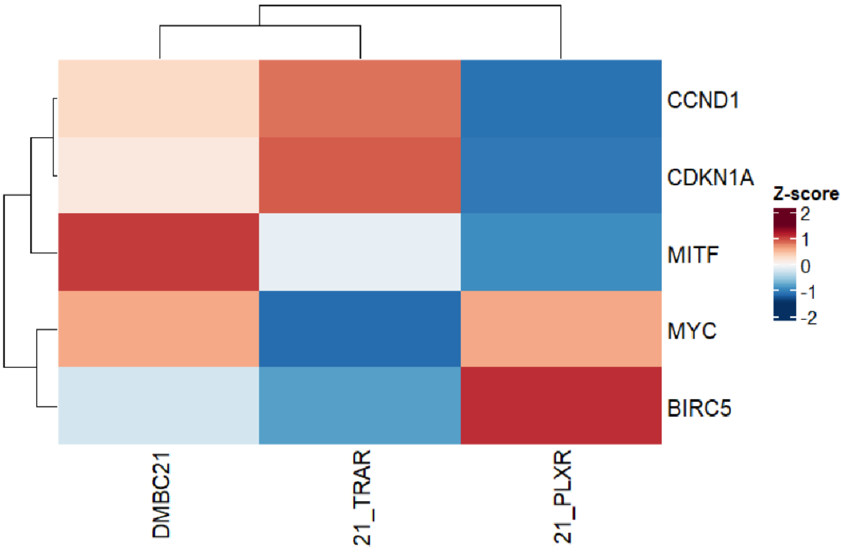

Figure S3. RNA-seq analysis of transcriptomic data from DMBC21, 21_TRAR, and 21_PLXR cells, submitted to GEO as the GSE301849 dataset. The FPKMs of *BIRC5*, *CCND1*, *MITF*, *CDKN1A,* and *MYC* are shown as z-scores, representing the relative expression of each gene across all three cell lines.
